# Improving Interlayer Interactions in Proton Exchange Membrane Fuel Cell through Carbon Nanotube‐Based Catalyst Design

**DOI:** 10.1002/advs.202514404

**Published:** 2025-11-14

**Authors:** Ling Ai, Zhen Xu, Stuart M. Holmes, Heng Zhai

**Affiliations:** ^1^ Department of Chemical Engineering The University of Manchester Oxford Road Manchester M13 9PL UK; ^2^ Department of Materials The University of Manchester Oxford Road Manchester M13 9PL UK

**Keywords:** catalyst layer design, CNT, interlayer interaction, PEMFC, sustainable energy

## Abstract

Proton exchange membrane fuel cells (PEMFCs) have gained significant attention due to their efficient use of hydrogen energy, high energy density, and zero emissions, making them ideal for future transportation and portable energy frameworks. Despite these advantages, challenges related to catalyst layer design, material durability, and cost‐effectiveness persist. This paper reviews recent advancements in the application of carbon nanotubes (CNTs) in PEMFCs. The study highlights the integration of CNTs at various levels of the membrane electrode assembly (MEA), focusing on both covalent and non‐covalent modification schemes. A particular emphasis is placed on vertically aligned carbon nanotube (VACNT) structures due to their potential to improve electron and mass transport pathways, leading to enhanced fuel cell performance and durability. Additionally, the synergistic effects of blending CNTs with carbon black (CB) are explored to address issues related to interlayer interactions and material conductivity. The review identifies gaps in current research, particularly in understanding the comprehensive impact of CNT modifications on the operational state of the MEA, and proposes new insights and strategies for material design aimed at facilitating the commercialization of PEMFC technology.

## Graphical abstract

This review highlights how carbon nanotube (CNT)–based catalyst designs enhance proton exchange membrane fuel cell (PEMFC) performance by improving interlayer interactions. Vertically aligned CNTs facilitate water management, mass transfer, and electron conduction while promoting effective active sites and reducing interfacial resistance. The integrative framework presented provides design principles for durable and efficient next‐generation PEMFC architecture.

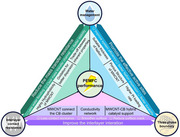

## 1 Introduction

Since the industrial revolution in the 1950s, the societal demand for energy has surged to unprecedented levels, provided largely by the burning of fossil fuels. This reliance on fossil fuels has precipitated a climate crisis. The National Oceanic and Atmospheric Administration (NOAA) recorded that 2023 was the hottest year since global records began in 1850, with temperatures 1.18 °C above the 20th‐century average of 13.9 °C.^[^ ^1^ ^]^ The recent Climate Change Conference in Glasgow (COP 26) also pointed out the urgent need for more decisive action.^[^ ^2^ ^]^ Proton exchange membrane fuel cells (PEMFC) are considered highly suitable for future transportation and portable energy systems, addressing climate issues and promoting sustainable development due to their efficient use of hydrogen energy, zero emissions, quick start‐up, and high energy density. The (U.S.) Department of Energy (DOE) has established a key performance‐cost index for the commercial PEMFC, requiring precious metal loading in the platinum group to be no more than 0.125 g kW^−1^.^[^ ^3^ ^]^ This means the MEA must achieve a power of 1 W cm^−2^ with a platinum loading of less than 0.125 mg cm^−2^. However, conventional MEAs generally require platinum loadings of 0.2 to 0.5 mg cm^−2^ to achieve a viable energy density.^[^ ^4^ ^]^ Technically, the commonly used carbon black (CB) catalytic support is suboptimal in terms of resistance to corrosion and platinum particle migration.^[^ ^5^ ^]^ The durability of fuel cell systems remains a significant barrier to commercial application. The two subtypes of PEMFC, low‐temperature PEMFC and high‐temperature PEMFC, are hampered by water management issues and MEA degradation.^[^ ^6^ ^]^ Therefore, optimizing the catalyst layer design is crucial for developing PEMFC technology, aiming for low‐cost and long‐term operation. Moreover, as PEMFC technology transitions from theoretical to practical engineering applications, catalyst layer design needs to achieve performance breakthroughs from new perspectives.

At this critical juncture in the development of PEMFC technology, solely optimizing the intrinsic structure of the catalyst layer is insufficient for meeting commercial requirements. A comprehensive structural approach should also consider the operational characteristics of the gas diffusion layer and the proton exchange membrane in the MEA.^[^ ^7^, ^8^, ^9^ ^]^ The interlayer functions of MEA can be summarized into four main areas: 1) mechanical adhesion, 2) interlayer electrical conduction, 3) interlayer mass transfer, and 4) interlayer heat transfer. Prolonged operation can cause local mechanical stripping of the MEA, destroy the proton conductor, and disrupt charge transfer. Effective interlayer electrical conduction requires low surface roughness and relatively uniform porosity to ensure good contact, thereby enhancing conductivity and reducing polarization between layers.^[^ ^10^, ^11^ ^]^ Regarding mass transfer, the complex humidity requirements and management across the catalyst layer, proton exchange membrane, and gas diffusion layer present challenges in optimizing interlayer performance. It is widely understood that the catalyst layer and the proton exchange membrane require moisture for effective proton conduction, while the gas diffusion layer typically needs to prevent obstruction of the flow channel caused by excess moisture. Poor interlayer contact, surface cracks, and water conduction mechanisms often lead to significant water accumulation at the catalyst layer/gas diffusion layer interface, which increases the oxygen transfer resistance. Finally, the thermal management of the MEA is primarily focused on the stack design, since the inability to dissipate the heat produced from the reaction can alter the water transport mechanisms and affect local catalyst performance. In summary, the four interlayer interactions of MEA are interconnected, and the optimal interlayer structure design should consider the functions and demands of all layers in an integrated manner. To date, advancements in materials science have resulted in a substantial number of modification strategies in catalyst layer fabrication, many of which have been theoretically proven to enhance catalytic performance. However, not all catalytic materials or modification schemes are appropriate for PEMFC in terms of its complex operation environment. Therefore, it is essential to evaluate the modification schemes based on engineering principles of interlayer interactions.

In the early stages of PEMFC technology development, the primary component within the catalyst layer was platinum black.^[^ ^12^, ^13^ ^]^ As the technology evolved, platinum black catalysts were replaced by supported catalysts due to their inherent shortcomings: a low specific surface area and poor utilization of active sites; insufficient catalytic activity and stability; high‐cost hindering commercialization. By dispersing platinum nanoparticles onto a high‐surface‐area carrier, the utilization rate, catalytic activity, and durability of platinum have been significantly enhanced. Furthermore, engineering strategies to modify catalyst supports with consideration of fuel cell operating conditions have enabled the design of interfacial interactions within MEA. To date, a variety of carbon‐based catalyst supports‐such as CB, carbon nanofibers (CNFs), graphene, carbon aerogels, CNT, and biomass‐derived carbons—have been reported.^[^ ^5^, ^14^, ^15^, ^16^ ^]^ Each of these carbon materials, however, exhibits certain limitations in practical applications. Conventional CB (e.g., mesoporous Vulcan XC‐72) offers high surface areas and good dispersibility but lacks long‐range conductive networks between particles. Efficient interlayer electron transport relies heavily on interfacial uniformity, while their abundant sp^3^ C‐C bonds are prone to corrosion and agglomeration under high‐potential cycling, ultimately causing structural collapse and performance degradation of the catalyst layer.^[^ ^17^ ^]^ The integration of CNFs with electrospinning techniques has attracted increasing attention in recent years, as it enables greater control over catalyst layer morphology and the fabrication of ordered structures.^[^ ^15^, ^18^ ^]^ CNFs provide good mechanical strength and partially porous channels; however, their surface areas are generally lower than those of CB, limiting platinum dispersion. Moreover, their relatively weak interfacial adhesion with ionomers in MEA hampers the formation of stable three‐phase boundaries, and their mass transport properties are inferior to those of CNTs with hollow tubular structures. Graphene can provide exceptional electrical conductivity and 2D morphology, but tends to stack during processing, which reduces the accessible surface area and impedes mass transport in the through‐plane direction.^[^ ^19^, ^20^ ^]^ Furthermore, the scalability of graphene synthesis and its processability during MEA fabrication remain significant challenges. Similarly, carbon aerogels and biomass‐derived carbons, though highly porous and synthetically versatile, often exhibit fragile frameworks and insufficient chemical stability under prolonged fuel cell operation.^[^ ^21^, ^22^ ^]^

CNTs are essentially formed by rolling graphene sheets into cylinders with a small diameter.^[^ ^23^ ^]^ Based on the number of concentric layers, CNTs can be classified into single‐walled carbon nanotubes (SWCNTs) and multi‐walled carbon nanotubes (MWCNTs). Since the discovery of CNTs by Iijima in 1991, the exploration of the mechanical properties, electronic structure, and chemical properties of CNTs has never stopped.^[^ ^24^ ^]^ At a time when the development of the PEMFC technology faces significant challenges, CNTs are anticipated to drive breakthrough advancements in the field. In addressing the key factors such as water management, low contact resistance, and the establishment of an effective three‐phase boundary for the MEA, CNTs show promise as a catalyst layer support. CNTs not only provide continuous pathways for electron and heat conduction within the catalyst layer but also create efficient gas and water transport channels across multi‐scale porous structures, thereby enhancing mass transport between layers. Their tubular morphology offers stable, well‐dispersed anchoring sites for platinum nanoparticles, improving platinum utilization and reinforcing the mechanical integrity of the catalyst layer. The robust sp^2^ C═C bonds confer high stability under oxidative environments and dynamic load conditions, improving catalyst durability. In addressing critical factors for MEA performance, such as water management, low interfacial resistance, and the formation of effective three‐phase boundaries, CNTs show exceptional promise as catalyst supports.^[^ ^25^ ^]^ However, the drawbacks of MWCNTs, such as strong agglomeration, wall surface chemical inertness, and unopened ends, are unfavorable for platinum nanoparticle loading, which is detrimental to MEA processing and leads to inhomogeneous catalyst layer surfaces. Consequently, this reduces the utilization of platinum and increases the contact resistance. Previous efforts have explored methods to modify CNTs, such as introducing polar functional groups through strong reflux oxidation conditions or incorporating nitrogen to create a localized pyridine structure.^[^ ^26^, ^27^ ^]^ However, excessive functionalization will compromise the favorable structure and properties of MWCNTs. Therefore, it is crucial to review recent MWCNT modification schemes and discuss these from the perspective of interlayer interactions in MEA to advance further applications of CNT in PEMFC.

**Figure 1:**
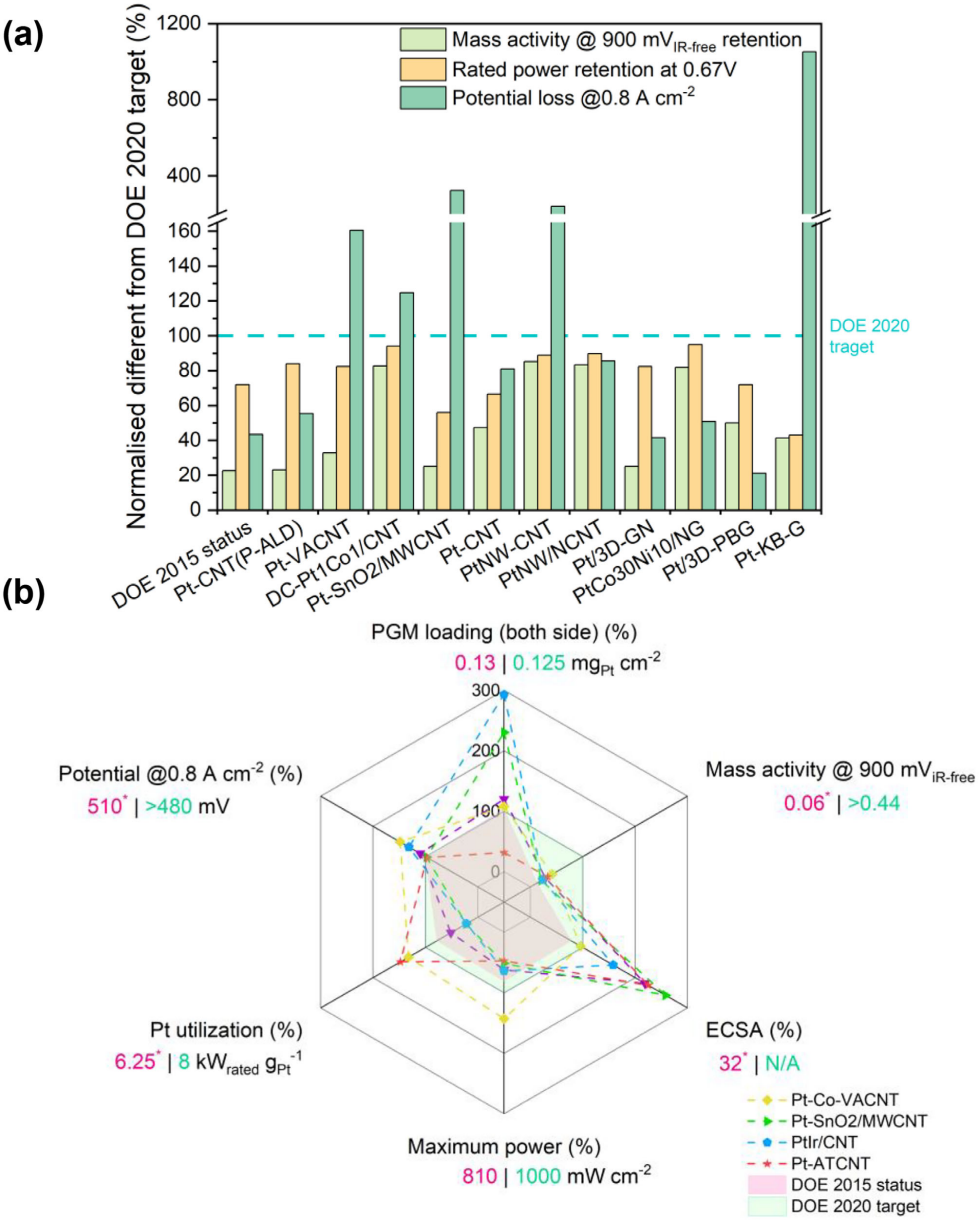
a) Normalized performance deviation of various advanced platinum‐based catalysts relative to the Department of Energy (DOE) 2020 technical targets.^[^ ^3^, ^28^, ^29^, ^30^, ^31^, ^32^, ^33^, ^34^, ^35^, ^36^, ^37^ ^]^ b) Radar chart comparing the multidimensional performance of selected catalysts.^[^ ^29^, ^31^, ^38^, ^39^ ^]^ While PGM refers to the platinum‐group metal; iR refers to the iR compensation is developed to correct for the voltage loss caused by the electrolyte solution between the working electrode and the reference electrode, where R is the resistance of the electrolyte solution; ECSA refers to the electrochemical surface area.

To underscore the urgency of catalyst redesign, recent comparisons between state‐of‐the‐art platinum‐based electrocatalysts and the DOE 2020 targets reveal that few existing systems achieve balanced performance across key parameters such as mass activity, potential loss, and power retention (**Figure** **1**a).^[^ ^3^, ^28^, ^29^, ^30^, ^31^, ^32^, ^33^, ^34^, ^35^, ^36^, ^37^ ^]^ Furthermore, radar chart analysis in Figure 1b shows that many advanced catalysts still struggle to meet multiple DOE benchmarks simultaneously, especially in platinum group metal (PGM) loading and utilization.^[^ ^29^, ^31^, ^38^, ^39^ ^]^ These data highlight the need for catalyst designs that can simultaneously optimize interlayer conductivity, oxygen diffusion, and structural durability (**Table** **1**). By comparing Figure 1 and Table 1, it is evident that during the initial stage of regeneration, most CNT‐based catalysts exhibit insufficient mass activity metrics. This reflects significant catalyst activation losses at 0.9 V for CNT‐based catalysts. Although a considerable number of studies have achieved peak powers exceeding the DOE target of 1000 mW cm^−2^, the relatively higher PGM loading results in insufficient platinum utilization at rated power. It is worth noting that CNT‐based catalysts demonstrate potential in terms of favorable electrochemical surface area (ECSA) and potential at moderate current densities (0.8 A cm^−2^). Regarding durability, although CNTs have demonstrated relatively good chemical stability, platinum particle migration and loss have prevented most approaches from meeting the commercial performance metrics set by the DOE. To the best of our knowledge, previous research has primarily focused on the application of CNT in each layer of PEMFC and explored numerous modification schemes. However, there has been less emphasis on the discussion of these modification schemes from the perspective of interlayer interactions and substantial consideration of the impact of the modification schemes on the overall operational state of the MEA. This oversight has led to a stagnation in practical engineering advancements. Therefore, this work aims to provide new insights into the application of CNT in PEMFC, particularly the employment of vertically aligned carbon nanotube (VACNT) that improves the electron and mass transport pathways for enhanced fuel cell performance and durability. Based on this, our goal is to offer subsequent researchers new ideas for PEMFC material design and facilitate the translation of PEMFC technology from theory to commercial applications.

**Table 1:** Performance metrics of various platinum‐based catalysts compared with the Department of Energy (DOE) 2015 baseline and 2020 targets.

Catalyst	Platinum‐group metal loading	Membrane	Mass activity @900 mV_i__R‐free_	Electrochemical surface area (ECSA)	Maximum power	Platinum utilization	Potential @0.8 A cm^−2^	References
	[mg_Pt_ cm^−2^]		[A mg_Pt_^−1^]	[m^2^ g_Pt_^−1^]	[mW cm^−2^]	[kW_rated_ g_Pt_^−1^]	[mV]
DOE 2015 status	0.13	N/A	0.06	32	810	6.250	514.058	[3]
DOE 2020 target	0.125	N/A	>0.44	N/A	>1000	8.000	N/A	[3]
Pt/SWCNT‐COOH	0.23	Nafion 212	0.25	57	592.59	2.576	627.780	[40]
Pt/CNT (P treated ALD growth)	0.4	Nafion 212	0.13	17.050	445.06	1.113	500.340	[28]
Pt/N‐MWCNT (MF resin treated)	0.15	Nafion 212	0.128	70	620	4.133	567.160	[27]
Pt‐Co/VACNT	0.135	Nafion 211	0.184	30.7	1430	10.593	764.290	[29]
DC‐Pt_1_Co_1_/CNT (2‐confinement)	0.06	Nafion 211	1.450	88.1	1260	21.000	411.220	[30]
Pt‐SnO_2_/MWCNT	0.288	Nafion 211	0.094	82.657	515	1.788	503.876	[31]
Pt/CNT	0.100	Gore 12 µm	0.152	61.7	1150	11.500	700.970	[32]
PtNW/CNT	0.3	Gore M740	0.094	59.6	1104	3.680	758.696	[33]
PtIr/CNT	0.367	Nafion 211	0.104	50.6	640.43	1.746	677.160	[38]
Pt/CNT	0.1	Nafion 211	0.134	15	836	8.360	543.730	[41]
PtNW/NCNT	0.4	Gore M740	0.187	81.3	966	2.415	743.590	[42]
Pt‐NiSA/CNT‐2.6	0.225	Nafion 211	0.059	73.2	1650	7.333	727.604	[43]
Pt_3_Fe‐ECNT (Etched)	0.15	Nafion 212	0.092	84	530	3.533	570.502	[44]
Pt/ATCNT	0.04	Nafion 211	0.146	72.06	473.1	11.828	499.599	[39]
Pt/3D‐GN	0.125	Nafion 211	0.362	22.76	1310	10.480	682.243	[34]
PtCo_30_Ni_10_/NG	0.133	Gore M820	0.492	71.661	866.7	6.517	708.571	[35]
Pt/3D‐PBG	0.14	Nafion 211	0.224	27.067	820	5.857	666.670	[36]
Pt/KB‐G	0.3	Nafion 212	0.025	30.128	839.19	2.797	501.35	[37]

## 2 Layers Design of PEMFC

### 2.1 Membrane Electrode Assembly Structure and Design

**Figure 2:**
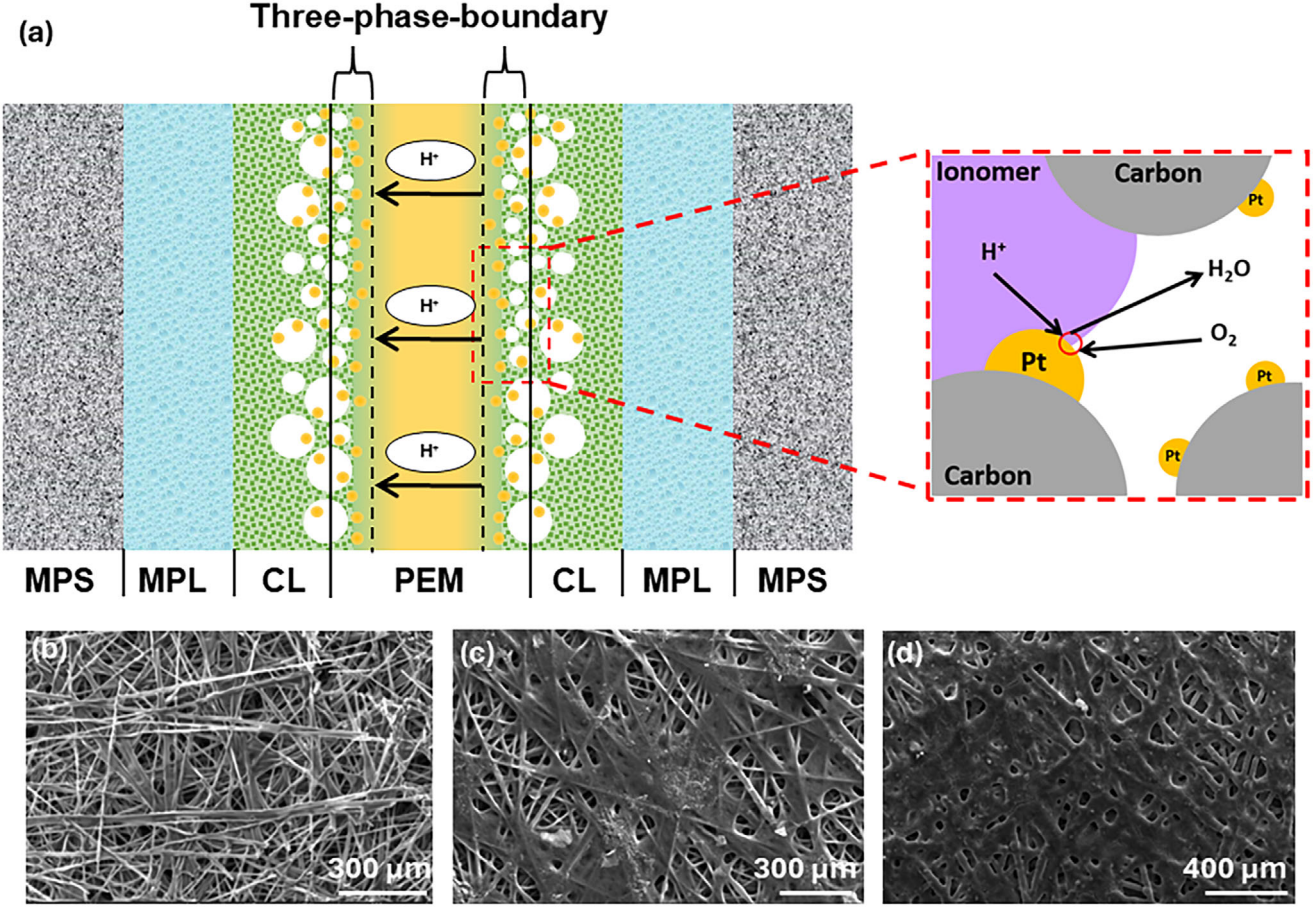
a) Schematic of the membrane‐electrode‐assembly structure and three‐phase boundary. MPL refers to the microporous layer, CL refers to the catalyst layer, and PEM refers to the proton exchange membrane; MPS refers to the macroporous support, which also known as gas diffusion support. b) Scanning electron microscopy (SEM) image of carbon paper (Toray TGPH 090, 280‐micron, Fuel Cell Store). c) SEM image of carbon paper coated with 1 mg_carbon_ cm^−2^ microporous layer ink. The ink was made with 90 wt% Ketjen black (EC‐300J, akzonobel) and 10 wt% polytetrafluoroethylene (60 wt% PTFE, Sigma Aldrich) in isopropanol (IPA, 99.50%, Fisher Scientific) dispersion. d) SEM image of an electrode coated with a catalyst layer. The catalyst layer contains 0.25 mg_Pt_ cm^−2^ commercial Pt/C catalyst with 60% platinum loading. After the catalyst layer coating, an additional layer of 0.5 mg cm^−2^ pure Nafion was coated onto the catalyst layer and acted as a binder between the catalyst layer and proton exchange membrane.

In PEMFC, the MEA is a fundamental component in the fuel cell system. A typical MEA structure, as illustrated in **Figure** **2**a, includes a proton exchange membrane flanked by two catalyst layers and two gas diffusion layer), each supported by a gas diffusion layer support. According to Andrew et al.’s book, these supports are also known as macroporous support and microporous layer. Figure 2b–d also reveals scanning electron microscopy (SEM) images of a typical carbon paper surface, as well as microporous layer and catalyst layer surfaces fabricated through a typical air spray process. The irregular texture of the carbon paper fibers and the presence of large voids lead to the uneven microporous layer and catalyst layer surfaces after spraying. This slightly compromises the contact between the electrode and proton exchange membrane, leading to inhomogeneous reaction and mass transfer. Nevertheless, the current MEA structure still facilitates the diffusion of fuel (hydrogen) and oxidant (oxygen) gases through the gas diffusion layer to the three‐phase boundary, where the electrochemical reaction takes place. This boundary is formed by the catalyst, the proton exchange membrane, and the ionomer electrolyte, as shown in Figure 2a. To understand the application of CNT's in MEA's, it is important to understand the importance of the different layers, their structures, and how they interact.

#### 2.1.1 Gas Diffusion Layer Structure

The gas diffusion layer situated between the flow‐field plate and the catalyst layer is a critical porous structure that performs multiple essential functions. Beyond ensuring uniform distribution of reactant gases and effective removal of product water, it also provides electronic conductivity, thermal conduction, and mechanical support to the catalyst layer. The gas diffusion layer is typically composed of a macroporous support made of carbon cloth and a microporous layer formed from fine carbon powders. In early gas diffusion layer designs (e.g., Toray's TGP‐H series), commercial carbon papers often exhibited pronounced variations in fiber length and diameter, which could result in the formation of local macropores on the interwoven carbon fiber surfaces. To address this, previous studies have explored the incorporation of CNT with carbon fibers to improve the flatness of the macroporous support.^[^ ^45^ ^]^ Maheshwari et al. further proposed blending heat‐treated MWCNT with carbon fibers to simultaneously enhance the electrical conductivity and hydrophobicity of carbon papers.^[^ ^46^ ^]^ Nevertheless, the use of high‐cost CNTs to enhance carbon paper performance is not economically viable. Moreover, a high percentage of doping complicates the gas conduction pathways within the carbon paper and increases mass transfer resistance.

To further optimize the matching between the gas diffusion layer and catalyst layer, researchers introduced a microporous layer onto the gas diffusion layer surface.^[^ ^47^ ^]^ The microporous layer is typically prepared from milled carbon powder and a binder (such as polytetrafluoroethylene), applied to the main carbon paper substrate via spraying or coating processes. Its fine pore structure significantly reduces mass transfer resistance for reactants, shortening the diffusion distance for gas molecules to reach catalytic sites. Concurrently, it plays a crucial role in preventing electrolyte leakage, enhancing hydrophobicity, and improving interfacial contact between the gas diffusion layer and catalyst layer. Furthermore, the microporous layer enhances thermal conductivity, thereby assisting the battery in maintaining a more uniform temperature distribution during operation. Recent research into catalyst layer/gas diffusion layer interlayer interactions has indicated that matching the pore sizes of the microporous layer and catalyst layer can improve contact and reduce interlayer resistance.^[^ ^48^ ^]^ Burheim et al. demonstrated the improvement in thermal conductivity provided by a microporous layer using X‐ray computed tomography (X‐CT) and SEM, showing that the microporous layer contributes to a more uniform temperature distribution across the electrode surface.^[^ ^49^ ^]^

Under operating conditions involving humidity and stress, issues such as gas diffusion layer pore blockage, delamination, and carbon material degradation all diminish fuel cell performance. In recent years, researchers have sought to further optimize gas diffusion layer performance through material structural design, for instance, by incorporating CNT into the microporous layer to enhance planarity and conductive architecture. Li et al. combined whisker‐like CNTs with Ketjen black to form a microporous layer. This crack‐free micronetwork structure exhibits reduced susceptibility to pressure variations, whilst its dense surface architecture enhances the MEA's water retention capacity and interlayer conductivity under low‐humidity conditions.^[^ ^50^ ^]^ The work by Kim et al. confirms that CNT flakes, whether employed as a microporous layer or as an additional layer in conventional gas diffusion layers, enhance the water retention and drainage properties of MEAs while significantly reducing charge transfer resistance (R_ct_).^[^ ^51^ ^]^ The scheme of fluorinating the CNT surface and then compositing it with CBs as a microporous layer further enhances the hydrophobicity and stability of the microporous layer under high humidity conditions.^[^ ^52^ ^]^ The CNT‐based microporous layer formed via processes such as thin‐film sandwiching or graded sedimentation creates a pore transition between the carbon fabric and the catalyst, thereby enhancing the interlayer coupling between the macroporous support and the catalyst layer.^[^ ^53^, ^54^, ^55^ ^]^ Owing to the excellent chemical stability of CNTs, the MEA maintains favorable electronic conductivity and structural stability over extended periods of operation, thereby achieving superior durability in Kwon et al.’s approach.^[^ ^56^ ^]^ Therefore, the pore size distribution, hydrophobic gradient, and compression stability of the gas diffusion layer not only determine the transport efficiency of gases and water but also influence the interfacial behaviour between the catalyst layer and the proton exchange membrane. For catalytic layer designs based on modified CNTs and vertically aligned nanostructures, a thorough understanding and optimization of the gas diffusion layer structure can provide more stable support and shorten mass transfer pathways, thereby maximizing their potential performance within the MEA.

#### 2.1.2 Catalyst Layer Structure

The catalyst layer is directly connected to the proton exchange membrane and is crucial for the performance and durability of the fuel cell system. The three‐phase boundary, consisting of the solid catalyst phase, the ionomer electrolyte phase, and the gas pore phase, is the site of the fuel cell reaction. The effective width of this boundary significantly influences oxygen transport resistance, ECSA, and charge transfer, all of which are vital for fuel cell performance. Structurally, the catalyst layer comprises a catalytic support and a catalytic centre metal. Current research on developing high‐performance catalysts and modifying catalytic supporting materials focuses on two main areas: alterations to the catalytic centre and modifications to the catalyst support material. Regarding the catalytic centre, platinum remains the only metal capable of meeting commercial performance standards. To reduce costs, various core‐shell structures have been developed that reduce platinum content by doping with less noble metals, though these have yet to yield satisfactory results. For catalyst support materials, a wide array of carbon‐based forms has been explored.^[^ ^57^ ^]^ CBs and CNTs are the predominant materials used for modifications. However, challenges such as platinum particle migration, aggregation, and the oxidative corrosion of support materials continue to hinder the fuel cell system's performance and durability.^[^ ^58^ ^]^ The structural design of the catalytic layer, including pore distribution, ionomer content, and platinum particle size and distribution, not only determines the reactivity and stability of the reaction interface but also influences the transport equilibrium between water and gases.^[^ ^32^ ^]^ For MEAs incorporating modified CNTs and vertically aligned nanostructures, such design optimizations enable the full exploitation of CNTs' high conductivity and the short‐range mass transfer advantages of vertically aligned structures. From the perspective of interlayer interactions, the design of the catalyst layer should not be confined solely to optimizing the material properties within the layer itself. It must also account for the coupling between the catalyst layer and both the proton exchange membrane and the gas diffusion layer. This is achieved by establishing clear and efficient pathways for mass and proton transport, thereby enhancing overall performance.

#### 2.1.3 Proton Exchange Membrane

In PEMFC systems, the proton exchange membrane primarily functions to transfer protons from the anode to the cathode while preventing the crossover of hydrogen and oxygen gases and blocking the direct passage of electrons through the membrane.^[^ ^59^, ^60^, ^61^ ^]^ Under operating conditions in fuel cells, the performance of proton exchange membranes depends not only on their intrinsic proton conductivity but also on their coupling with other functional layers, such as the gas diffusion layer and catalyst layer. Whether CNTs are incorporated into the membrane material itself to enhance structural integrity or employed as an intermediate material to indirectly influence the proton exchange membrane's water management, a thorough understanding of the proton exchange membrane's characteristics and operational mechanisms is essential to optimize interactions between the proton exchange membrane and other functional layers.

**Figure 3:**
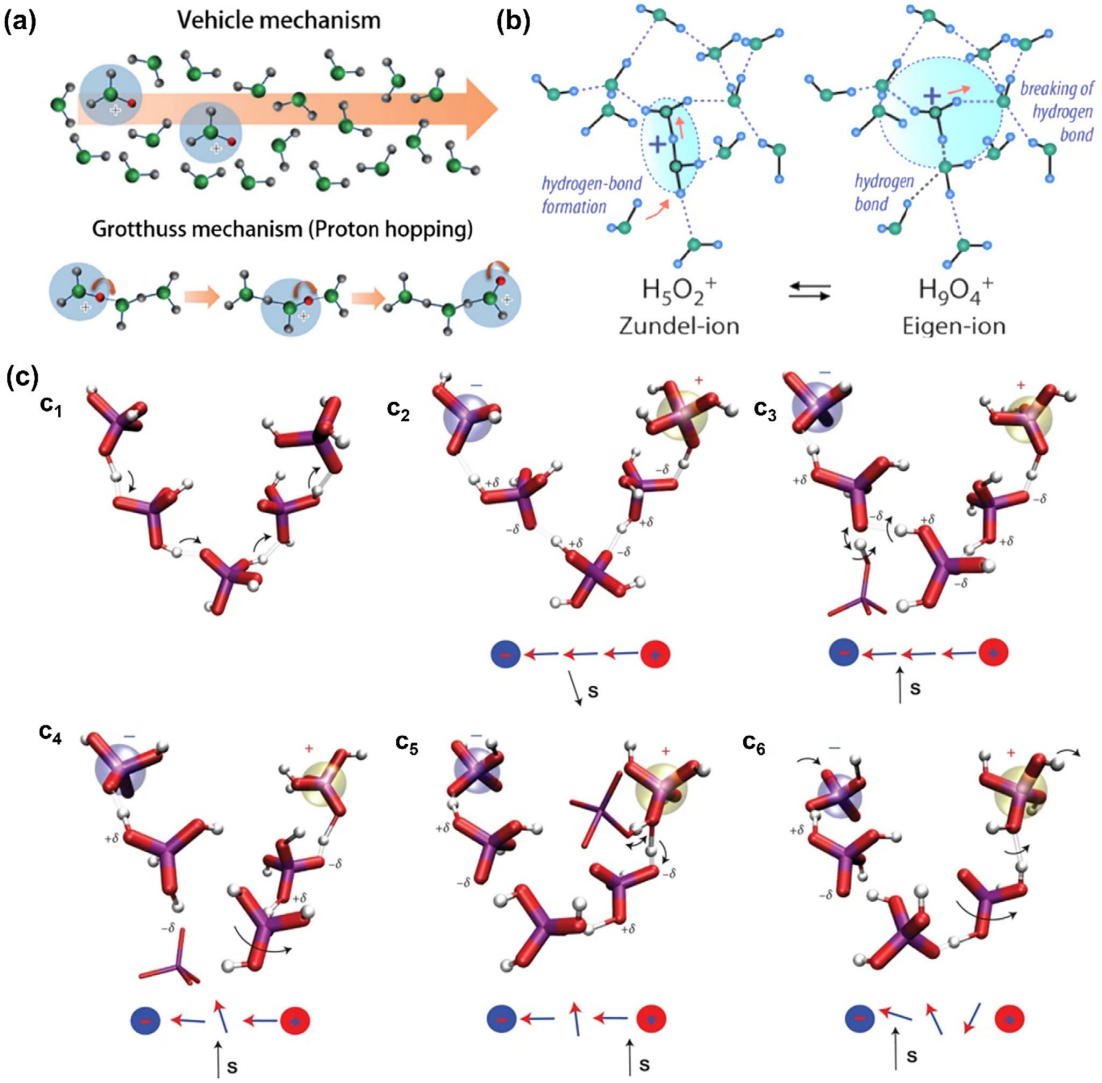
a) The schematic design of the vehicular mechanism and hopping mechanism for proton transfer in low‐temperature proton exchange membrane fuel cell. b) Proton transport in water via hydrogen bond in high‐temperature proton exchange membrane fuel cell. Reproduced with permission.^[^ ^62^ ^]^ Copyright 2024, Wiley. c) The Grotthuss mechanism of proton transfer in phosphoric acid for high‐temperature proton exchange membrane fuel cell. It illustrates proton conduction in H_3_PO_4_ via the **Grotthuss mechanism** (white, hydrogen; red, oxygen; purple, phosphorus), where protons transfer through hydrogen‐bonded phosphate molecules. **Correlated proton hopping** (c_1_, c_2_) forms transient chains, followed by **solvent relaxation** (c_3_) and hydrogen bond reorganization (c_4_). This enables continuous **charge separation and proton migration** (c_5_, c_6_), ensuring efficient conductivity in high‐temperature proton exchange membrane fuel cell at high temperatures. Reproduced with permission.^[^ ^63^ ^]^ Copyright 2012, Springer Nature.

The Nafion, developed by DuPont, is a perfluorosulfonic acid (PFSA) polymer generated by free radical‐initiated copolymerization of a perfluorinated vinyl ether sulfonyl fluoride co‐monomer with tetrafluoroethylene (PTFE), which is commonly used in low‐temperature proton exchange membrane fuel cells (LT‐PEMFCs). There are two main mechanisms of proton migration in LT‐PEMFCs: the vehicular mechanism and the Grotthuss mechanism (also called the hopping mechanism), as shown in **Figure** **3**a.^[^ ^62^ ^]^ Proton transfer in sulfonic acid matrices is influenced by hydration state: under high humidity conditions, the Grotthuss transition mechanism predominates due to the formation of continuous hydrogen‐bond networks, whereas under low humidity conditions, the vehicular mechanism becomes dominant. In the bulk phase, the Grotthuss mechanism promotes the formation of hydrogen bonds between protons and adjacent water molecules, leading to the creation of an H_5_O_2_^+^ dimer. This dimer decreases the average bond length within the hydrogen bond, facilitating proton migration to a second oxygen‐containing centre. The subsequent formation of the H_9_O_4_^+^ intermediate induces the breakage of hydrogen bonds, releasing the proton. This interconversion between the H_5_O_2_^+^ dimer and the H_9_O_4_^+^ intermediate allows the proton to “jump” within the hydrogen bonding network of water molecules (Figure 3b). In surface diffusion, the hydrophilic sulphonic acid groups enhance the movement of water molecules between these groups, aiding in the proton transfer across the membrane. The practical application of Nafion membranes faces two main challenges: the costly fluorination step in synthesizing the ionomer and the potential for dehydration and failure of the membrane at high temperatures, including a glass transition phase. Therefore, for high‐temperature PEMFC systems, the development of durable and performance‐stable proton exchange membrane materials is the technological bottleneck that needs to be overcome.

When examining how CNTs enhance interlayer interactions within the MEA, it is important to comprehend the conductive properties of the proton exchange membrane at varying operating temperatures, particularly in the context of high‐temperature proton exchange membrane fuel cells (HT‐PEMFCs), which have garnered significant attention in recent years. Alterations in proton conduction mechanisms directly influence humidity distribution, charge migration, phosphate migration, and structural stability at the proton exchange membrane/catalyst layer interface. Consequently, these factors indirectly determine whether the advantages of CNTs or vertically aligned nanostructures within the MEA can be effectively realised. According to the literature, as shown in Figure 3c, proton transport in H_3_PO_4_ occurs through correlated proton transfers along a network of hydrogen bonds, forming transient **Grotthuss chains**.^[^ ^63^ ^]^ This mechanism involves sequential proton hopping, where a proton is transferred from one phosphate molecule to another via rapid hydrogen bond rearrangement. The process is facilitated by solvent relaxation, which helps redistribute charge and stabilize newly formed hydrogen bonds. As described, proton transfer is not merely a simple diffusion process but is instead coupled with molecular reorientations that enable continuous charge migration. The independence of this migration from long‐range solvent rearrangements ensures efficient proton conduction, a critical factor in maintaining the high ionic conductivity required for HT‐PEMFC performance. Therefore, focusing on high‐temperature proton conduction within phosphoric acid systems, the conductive framework of CNTs serving as a catalyst layer or gas diffusion layer must be designed to accommodate both the membrane's operating environment and conduction mechanisms. This ensures stable interlayer interface behaviour and overall performance under varying temperature and humidity conditions.

In the early stages of PEMFC technology research, investigations into proton exchange membranes encountered mechanical strength issues when pursuing thinner thicknesses. Membrane rupture caused by swelling and stress fatigue under fuel cell operating conditions constrained progress in this direction. Researchers attempted to incorporate CNTs as a reinforcing framework within the proton exchange membrane to enhance mechanical strength. However, the long‐axis structure and high electrical conductivity of CNTs often resulted in lower permeability thresholds, subsequently triggering proton exchange membrane short‐circuiting.^[^ ^64^ ^]^ In terms of proton conductivity, non‐functionalized CNTs exhibit no proton‐conducting capability, and the deterioration in proton conduction caused by doping reduces the performance of PEMFCs. It should be noted that proton conduction within sulfonic acid proton exchange membranes is not governed by a single mechanism but is regulated by hydration states. Consequently, achieving stable and efficient proton transport within the MEA depends not only on the membrane's own microstructure but is also influenced by the distribution of water and the hydrophilic‐hydrophobic gradient at the interfaces between the catalyst layer and gas diffusion layer. The stable chemical properties of CNTs and the well‐defined mass transfer pathways within their vertically aligned structures provide a foundation for predicting the degree of hydration in proton exchange membranes and investigating the distribution of proton conduction flux.

### 2.2 Catalyst Layer Design in Fuel Cell

PEMFC technology is undoubtedly a promising method for energy conversion, and the maturation of MEA technology indicates a shift from theoretical research to commercialization. However, the performance and stability of current PEMFCs are not only constrained by catalytic materials but also by engineering challenges such as high oxygen mass transfer resistance at low platinum loading, flooding issues in low‐temperature PEMFC, and poor interlayer contacting, all of which impede performance improvement. The design of catalyst layers in PEMFCs primarily focuses on optimizing the materials within the layer, either by enhancing the catalytic activity centres or by refining the morphology of the catalyst supports to boost catalytic performance.

A series of review works has summarized the current catalyst layer design from various perspectives. Borup et al. discussed in situ and ex‐situ durability testing methods, highlighted the optimization of electrode stability and catalyst particle resistance to oxidative decomposition to improve durability, and proposed the use of low beginnings‐of‐life transition metal doping to retard degradation.^[^ ^65^ ^]^ Similarly, Zhao et al. focused on the endurance issue in terms of the corrosion mechanisms of the catalyst support, highlighting the influence of carbon support morphology through adjustments in the degree of graphitization and surface functionalization.^[^ ^5^ ^]^ Considering the high cost of platinum, Banham et al. reviewed the progress in precious metal‐free catalysts for PEMFC, revealing design limitations and noting that noble metal‐free solutions might also incur higher costs in small‐scale production.^[^ ^66^ ^]^ Du et al. highlighted the compromises in catalyst layer thicknesses within noble metal‐free designs.^[^ ^67^ ^]^

The current focus of catalyst reviews on PEMFC still predominantly centres on different catalyst supports. For example, Uchida's work emphasized the impact of the porosity of the support on the dispersion of ionomers and oxygen mass transfer.^[^ ^68^ ^]^ The work of Mohideen et al., Ortiz‐Herrera et al., Kwon et al., and Peera et al. reviewed CNT‐based catalytic strategies, including synthesis and modification approaches.^[^ ^15^, ^69^, ^70^ ^]^ Historically, the design of catalyst layers has been confined to internal optimizations, with less consideration given to how these designs affect other functional layers. Interactions within the MEA during operation, such as moisture produced in the cathode catalyst layer, which can enhance proton conductivity in the membrane but may also obstruct gas mass transfer channels in the gas diffusion layer, highlight the importance of interlayer interactions. These interactions, driven by humidity and heat accumulation as well as by the contacts between layers, influence each functional layer's effect on others. To the best of our knowledge, the design concepts that integrate interlayer interactions remain limited. This work aims to offer new perspectives and insights into the design of PEMFCs, supporting the advancement towards commercialization goals of PEMFC technology.

### 2.3 Interlayer Conductivity

The performance of MEA is significantly influenced by electron and proton transport between layers and the contact resistance. On a macro level, the interlayer conductivity in MEA is mainly influenced by the process of interlayer bonding. Factors impacting this process include the degree of mechanical compression,^[^ ^71^ ^]^ the assembly method of the MEA,^[^ ^72^ ^]^ the design of the electric bipolar plate,^[^ ^73^, ^74^ ^]^ and the material used in each layer.

Ge et al. studied the impact of varying the torque used to clamp MEA screws on the compression of the gas diffusion layer and the subsequent changes in MEA performance.^[^ ^71^ ^]^ Results revealed that cell performance decreases with increasing compression, with more significant performance degradation under conditions of over‐compression and low reactant flow, where an optimum compression ratio is crucial. Similar studies have highlighted the issue of uniformity of compression on gas diffusion layer surfaces, where an uneven degree of compression will lead to variations in local charge transport and uneven corrosion problems.^[^ ^75^ ^]^ The study of the compression effort on the gas diffusion layer has been investigated both experimentally and by computational fluid dynamics (CFD), with emphasis on the optimum compression ratio and stress uniformity at the electrode surface. Although research exploring the impact of compression on the proton exchange membrane/catalyst layer interface is still sparse, it generally indicates that inappropriate force levels can detrimentally affect gas diffusion layer performance. Additionally, electric bipolar plates, typically made of metal or graphite, are a significant focus of current research, especially regarding the design of flow field shapes.^[^ ^76^, ^77^ ^]^

**Figure 4:**
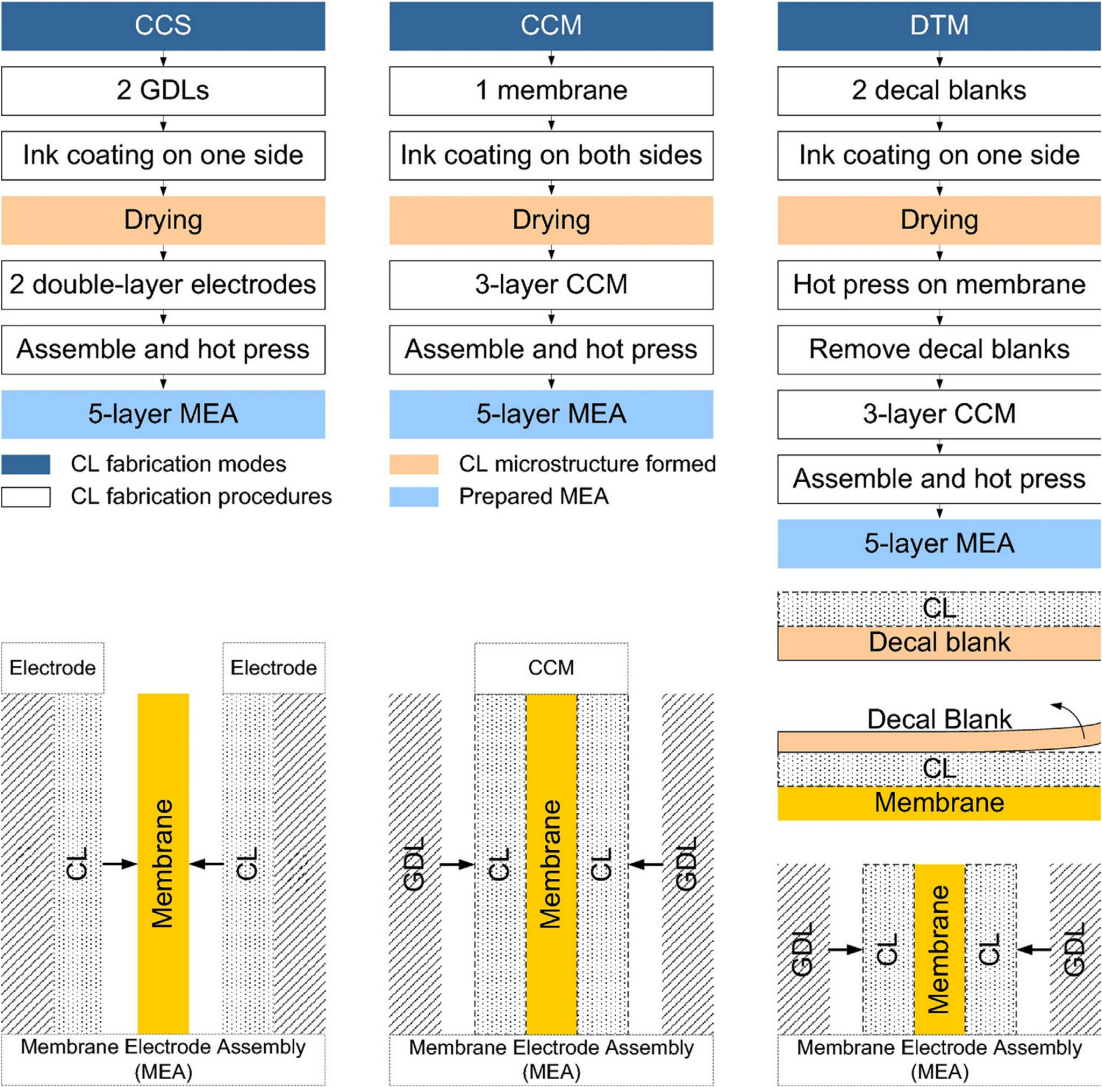
Schematic of catalyst‐coated membranes (CCM) method, catalyst‐coated substrate (CCS) method, and decal transfer (DT) method. Reproduced with permission.^[^ ^86^ ^]^ Copyright 2023, Springer Nature.

In terms of assembly methods, the generally accepted MEA assembly approaches include catalyst‐coated membrane (CCM) and catalyst‐coated substrate (CCS), and decal transfer (DT) method, which depend on different catalyst layer loading techniques.^[^ ^78^ ^]^ **Figure** **4** illustrates the process of these three assembly methods. The CCS method is relatively straightforward and offers potential for large‐scale MEA production. However, it often necessitates higher platinum loadings to ensure effective distribution of platinum particles on the electrode surface, and it also incurs additional losses due to the spraying process. The macroscopic disordered structure resulting from catalyst ink spraying hinders precise control over the interlayer structure of the MEA, and poor bonding at the thermo‐compression bonded catalyst layer/proton exchange membrane interface can cause localized conductivity loss, which is detrimental to long‐term operation.^[^ ^79^ ^]^ Conversely, the CCM method employs strategies that differ fundamentally from CCS. Popular techniques include direct spraying onto the proton exchange membrane (CCM‐DS) or using the decal transfer technique (CCM‐DT) for ex‐situ catalyst layer preparation.^[^ ^80^ ^]^ Thanasilp et al. demonstrated that the CCM‐DT method achieved ECSA values 1.76 and 1.05 times greater than those of the CCS method and the CCM‐DS method, respectively.^[^ ^80^ ^]^ The superior performance of the CCM method is attributed to a better combination of the catalyst layer and proton exchange membrane. The advantages of CCM are also reflected in the lower platinum loading and the more controllable catalyst layer morphology. Most of the macroscopically ordered catalyst layer structure schemes reported to date employ nanotube forest structures, which require the transfer of catalysts for ex‐situ synthesis using the CCM‐DT method.^[^ ^81^, ^82^, ^83^, ^84^ ^]^ However, the CCM method has the disadvantage that the gas diffusion layer bound by hot pressing tends to separate during long‐term operation and deteriorate the contact between the gas diffusion layer and the catalyst layer, thereby compromising the durability of the fuel cell system.^[^ ^85^ ^]^ Regardless of whether the MEA is manufactured using the CCS or CCM method, the problem of poor interlayer contact resulting from hot pressing is significant and underscores the necessity of exploring ways to optimize catalyst layer surface morphology and develop novel material design ideas for improved layer integration.

**Figure 5:**
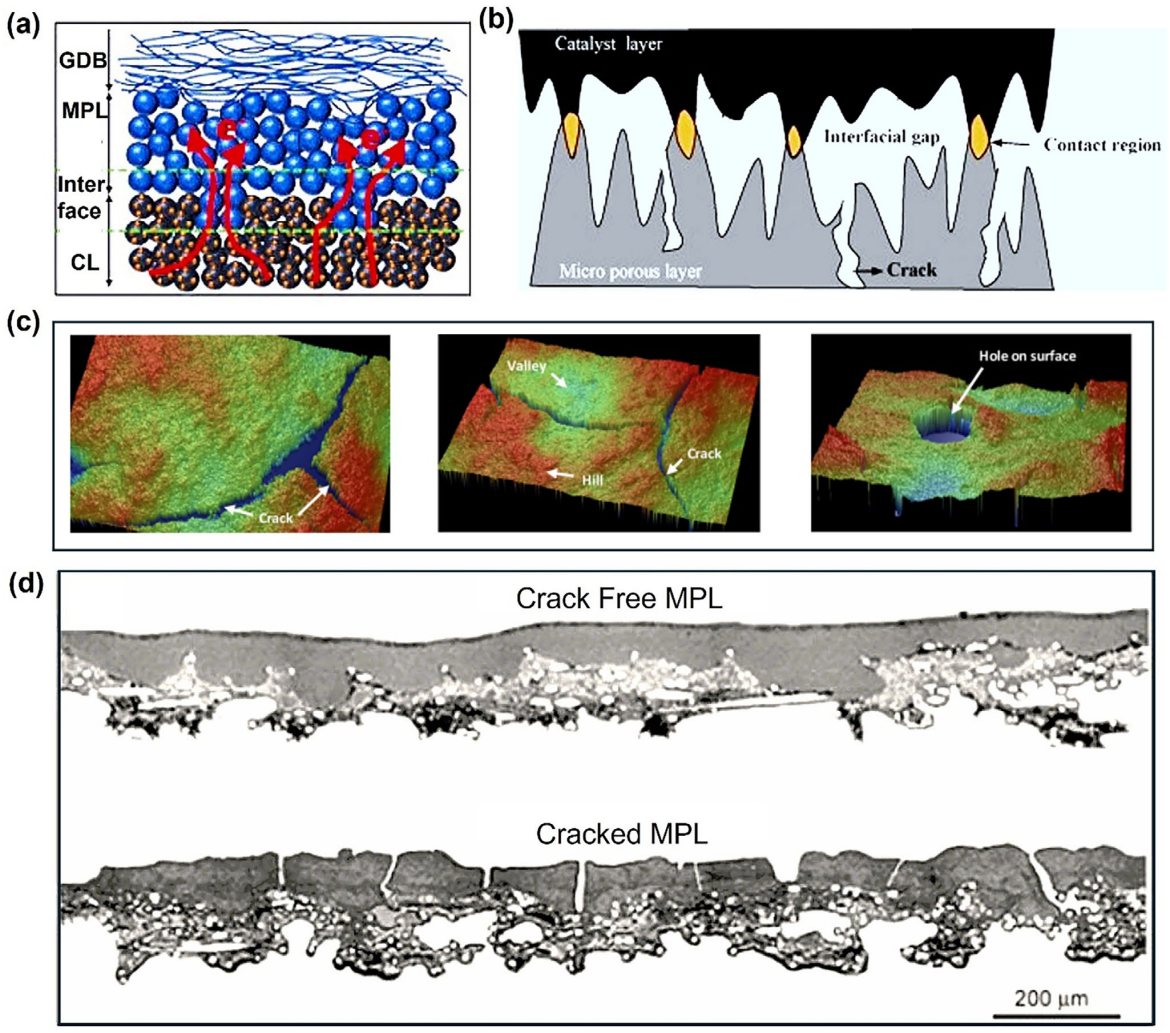
a) Schematic of electron transport at the catalyst layer/microporous layer interface.^[^ ^88^ ^]^ While GDB refers to the gas diffusion backboard, which is known as gas diffusion support or macroporous layer in this work. Reproduced with permission. Copyright 2024, Elsevier. b) Schematic of the imperfect contact of the microporous layer and catalyst layer in a cross‐sectional view. c) Morphology of microporous layer and catalyst layer surfaces by optical profilometry. Reproduced with permission.^[^ ^89^ ^]^ Copyright 2010, Elsevier. d) SEM micrographs of cross‐sectional cracked and crack‐free regions in the microporous layer.^[^ ^90^ ^]^ While MPL refers to the microporous layer. Reproduced with permission. Copyright 2010, IOP Science.

At the microscopic level, the surface morphology and roughness of the catalyst layer and gas diffusion layers are core properties that influence the contact resistance at the catalyst layer/gas diffusion layer interface. The microporous layer has been proven to have a performance‐enhancing effect on MEAs and has evolved into the prevailing standard for MEA manufacturing.^[^ ^87^ ^]^ The tighter contact in the imprinted region between the catalyst layer and microporous layer helps reduce the interface resistance and contributes to the charge transport, as illustrated in **Figure** **5**a.^[^ ^88^ ^]^ A characterization study of the microporous layer/catalyst layer interface by Hizir et al. pointed out the effect of microporous layer and catalyst layer surface smoothness and cracks on interlayer contact resistance and mass transfer (Figure 5b,c).^[^ ^89^ ^]^ Imperfect contact will lead to localized detachment and loss of electrical conductivity, as shown in the SEM micrographs of cross‐sectional cracked and crack‐free regions in a microporous layer (Figure 5d).^[^ ^90^ ^]^ The effect of macroscopic mechanical compression on reducing the surface roughness of the material is very limited. Furthermore, their work indicated that the microporous layer with a thickness of 80 µm can retain 0.22 to 0.71 mg cm^−2^ of water in surface cracks, and this water accumulation further impedes the interlayer conductivity. For improving interlayer conductivity and addressing poor contact, two approaches have been pursued in engineering the morphology of the catalyst layer and microporous layer interface: developing microporous layer and catalyst layer with ultra‐smooth surfaces^[^ ^91^, ^92^ ^]^ and fostering the in situ growth of the catalyst layer on microporous layer,^[^ ^93^, ^94^ ^]^ with the latter being preferred for enhancing conductivity due to its integral structure. Han et al. suggested that the increase of carbon loading in the microporous layer facilitates the construction of a smoother surface and reduces the contact resistance of the gas diffusion layer/catalyst layer, though a thicker microporous layer can hinder mass transfer.^[^ ^95^ ^]^ The in situ growth of the catalyst layer on the microporous layer is currently a focal point for developing a macroscopically ordered catalyst layer based on nanotube arrays. However, due to constraints associated with vapour phase chemical vapour deposition (CVD) and substrates, most projects resort to ex‐situ growth and the CCM‐DT method.^[^ ^81^, ^83^, ^84^ ^]^ The challenge of controlling a vertically aligned morphology for nanotube arrays and the limitations of the growth mechanism curtail the realization of this ideal.

The influence of ionomers on the performance of the proton exchange membrane/catalyst layer interface is reflected in both proton conductivity and reactant conduction resistance. Lee et al. implied in their work that in platinum supported on carbon (Pt/C) catalyst, the most widely‐used catalysts, only a small number of particles can be distributed in regions with suitable ionomer thickness.^[^ ^96^ ^]^ A thin ionomer coating will limit proton conduction, and an excessively thick ionomer coating will limit reactant diffusion to the platinum particle surface, increasing the resistance to gas transport. Additionally, the impact of moisture distribution in the ionomer and catalyst layer has been further studied by Malek et al.^[^ ^97^ ^]^ At low platinum content, the ionomer side chains tend to favor the outer gas diffusion support (macroporous layer), and the catalyst layer zone tends to be hydrophobic. At high platinum content, the side chains tend to favor the catalyst layer. Although the hydrophilicity of the catalyst layer is enhanced and water can also act as a proton conductor, the resistance to mass transfer is also enhanced. They suggested that the ratio between the porous structure content of the catalyst layer carbon support and the ionomer‐to‐carbon ratio (I/C) is a key indicator of the ionomer‐catalyst properties. Lowering the I/C ratio (around I/C = 0.375) facilitates higher limiting current density; increasing the I/C ratio (around I/C = 0.75) facilitates higher maximum power density and proton exchange membrane film wetting.^[^ ^97^ ^]^ The modification strategies for the proton exchange membrane are mainly through patterning the membrane surface and increasing the contact area.^[^ ^98^, ^99^, ^100^ ^]^

### 2.4 Interlayer Mass Transfer and Water Management Improvement

Mass transfer across interfaces involves the transport of reaction gases and moisture, determining the high current density operational performance of LT‐PEMFC. Within the gas diffusion layer/catalyst layer interface, the hollow tubes of CNTs provide a high‐speed molecular channel. With inner diameters typically ranging from 8 to 20 Å, the molecularly smooth and chemically inert inner walls enable gas molecules or small molecules to exhibit ultrafast, quasi‐ballistic flow. This flow velocity far exceeds predictions from classical Hagen–Poiseuille or Knudsen diffusion models.^[^ ^101^ ^]^ Within CNTs of sub‐nanometre diameter, gases or molecules are compelled to adopt a one‐dimensional linear arrangement. This reduces intermolecular interactions and enhances transport rates. For small molecules such as water and hydrogen, as well as other light gases, an anomalous isotope effect manifests (where the mass flow rate of H_2_ exceeds that of D_2_). This indicates the presence of quantum fluctuations and specular reflection effects, further elevating the permeation rate of light molecules.^[^ ^101^ ^]^ In PEMFCs, when CNTs are assembled into thin films or scaffold structures, naturally formed multiscale pores ranging from nanometres to micrometres emerge between the tubes. The large/mesopores provide the primary gas transport pathways, reducing macroscopic diffusion resistance, while the micropores and hollow channels enhance gas‐surface interactions. The quantum effects within CNT pores synergize with the voids formed by CNT tube stacking, significantly reducing gas transport resistance both within and between catalyst layers.^[^ ^101^ ^]^ Compared to conventional CBs or graphite flakes, the continuous channels within CNT structures minimize dead zones and local concentration polarization, proving particularly crucial for enhancing gas accessibility within the oxygen reduction reaction (ORR) zone.

The work of Song et al. reports the achievement of power reversal growth in fuel cells under high current regions by adjusting the proportion of whisker‐like CNTs within the CB‐based microporous layer.^[^ ^102^ ^]^ Their study indicates that large‐scale cracks (>10 µm) are readily filled by liquid water under high humidity conditions, forming interconnected water films that impede oxygen diffusion. This leads to concentration polarization and diminished power output. The CNT rigid and spatial framework reduces the number and width of large cracks while introducing medium‐scale cracks (5–10 µm). These cracks provide independent capillary pathways, whilst smaller pores remain available for gas diffusion, enabling separate gas and liquid transport and alleviating mass transfer resistance.^[^ ^102^ ^]^ Liu et al. synthesized ultrathin porous CNTs featuring 2–3 nm wall thicknesses, which formed a self‐supporting reticular catalytic layer less than 1 µm thick.^[^ ^103^ ^]^ The abundant sites on the support surface and excellent chemical stability enabled this approach to simultaneously meet the mass activity requirements for beginning‐of‐life and the DOE criteria for load variation and start‐stop cycle durability. The influence of CNT blending on mass transfer extends beyond its unique structure; alterations in the rheological properties of the catalyst ink impact the final deposition morphology and mass transfer at the electrode surface. Mehrazi et al. noted that the 20 wt.% CNT‐doped sample exhibited a higher average low‐shear viscosity, indicating rheological characteristics associated with a less compact microstructure featuring larger agglomerates. Inks containing pure CB nanoparticles exhibited a more compact aggregation network, reducing the accessibility of catalytic sites.^[^ ^104^ ^]^

**Figure 6:**
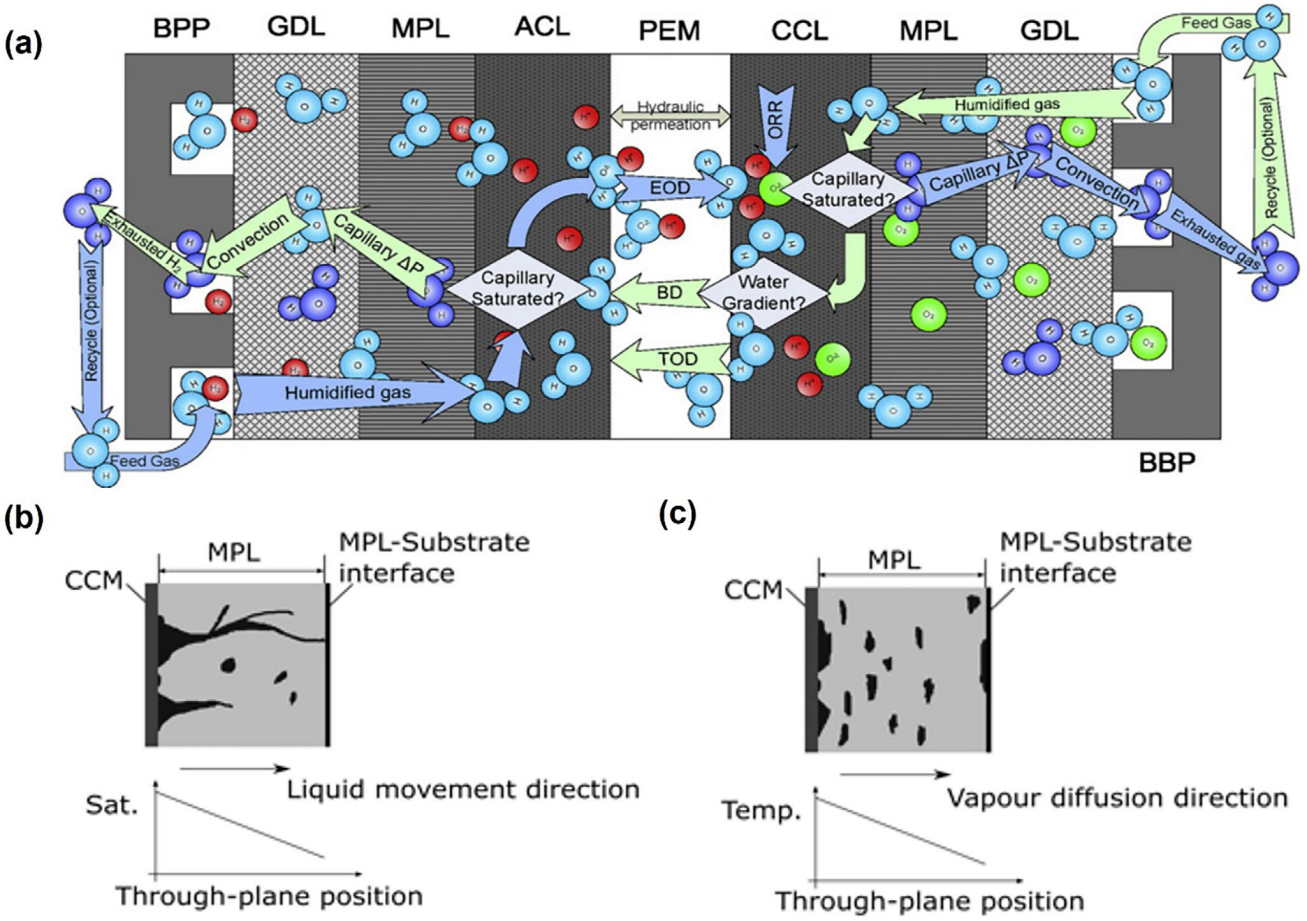
a) Schematic of the water transport pathway in the MEA. BPP refers to electric bipolar plates, ACL and CCL refer to the catalyst layer in the anode zone and cathode zone, EOD refers to electro‐osmotic drag, BD refers to back diffusion, and TOD refers to thermal‐osmotic drag. Reproduced with permission.^[^ ^109^ ^]^ Copyright 2009, Elsevier. b) Schematic and water saturation plot for the liquid phase water breakthrough mechanism. c) Schematic and water saturation plot for the vapour phase diffusion mechanism. Reproduced with permission.^[^ ^106^ ^]^ Copyright 2017, Elsevier.

Significant water management issues at high current densities frequently compromise interfacial mass transfer, with accumulated liquid obstructing gas transport pathways and disrupting the sustained, uniform delivery of reactants to active sites. The unique high aspect ratio pore structure of CNTs facilitates interlayer gas diffusion, while their pronounced hydrophobicity is pivotal for ameliorating PEMFC water management challenges and enhancing moisture transport at elevated current densities—both being essential for achieving superior interlayer mass transfer. **Figure** **6**a presents a water transfer mechanism in MEA. Water transport in the MEA is influenced by four main driving forces: electro‐osmotic drag (EOD), back diffusion (BD), the capillary effect, and pressure‐driven hydraulic permeation. The EOD is linearly related to the current density, allowing a fixed number of water molecules in the humidified gas to migrate from the anode to the cathode via the proton exchange membrane.^[^ ^105^ ^]^ Such a mechanism can be used to supplement water removal, which is beneficial from the phase change and the capillary effect. Due to the temperature gradient in the MEA, the EOD causes the water to migrate towards the colder side, which is more significant at higher temperatures (> 80 °C). Finally, there is also pressure‐driven hydraulic permeation in the proton exchange membrane, which conducts water in both directions, but is not generally considered to be the primary factor of water transport within the MEA. In the microporous layer of PEMFC, the capillary effect is the only feasible approach to remove water from the microporous layer and is ultimately removed by the gas flow. Unfortunately, the removal of water by capillary effect is highly dependent on the degree of water saturation in the microporous layer, which is very limited at high humidity and further results in significant water accumulation at the gas diffusion layer/catalyst layer interface. For high‐temperature PEMFC, the removal of water can also be achieved by evaporation phase change, which greatly simplifies water management issues compared to low‐temperature PEMFC. Ge's team modelled water transport within the microporous layer, highlighting two transport mechanisms for water in the microporous layer: liquid‐phase breakthrough (Figure 6b) and gas‐phase diffusion (Figure 6c).^[^ ^106^ ^]^ In liquid phase transport, liquid water penetrates and saturates the pores as the liquid phase pressure increases beyond the threshold capillary pressure of the adjacent pores. The penetration of liquid‐phase water into the surrounding pores gradually forms a pathway from the electric bipolar plate to the gas diffusion layer/catalyst layer interface. Following the breakthrough event, all liquid‐phase water is transported exclusively through this established pathway. No significant relationship between the subsequent liquid water transport and the pore structure of the rest of the microporous layer.^[^ ^106^, ^107^, ^108^ ^]^ For gas phase moisture transfer, the water vapour concentration gradient is the main driving force, with higher humidity at the catalyst layer on the hydrophilic side. For the gas phase moisture transport mechanism, the phase change of moisture condensation due to temperature changes is also a non‐negligible factor leading to flooding.^[^ ^106^ ^]^ This work further highlighted a higher moisture content at the microporous layer/catalyst layer interface on the cathode side and a higher saturation at the high system operating temperatures.

**Figure 7:**
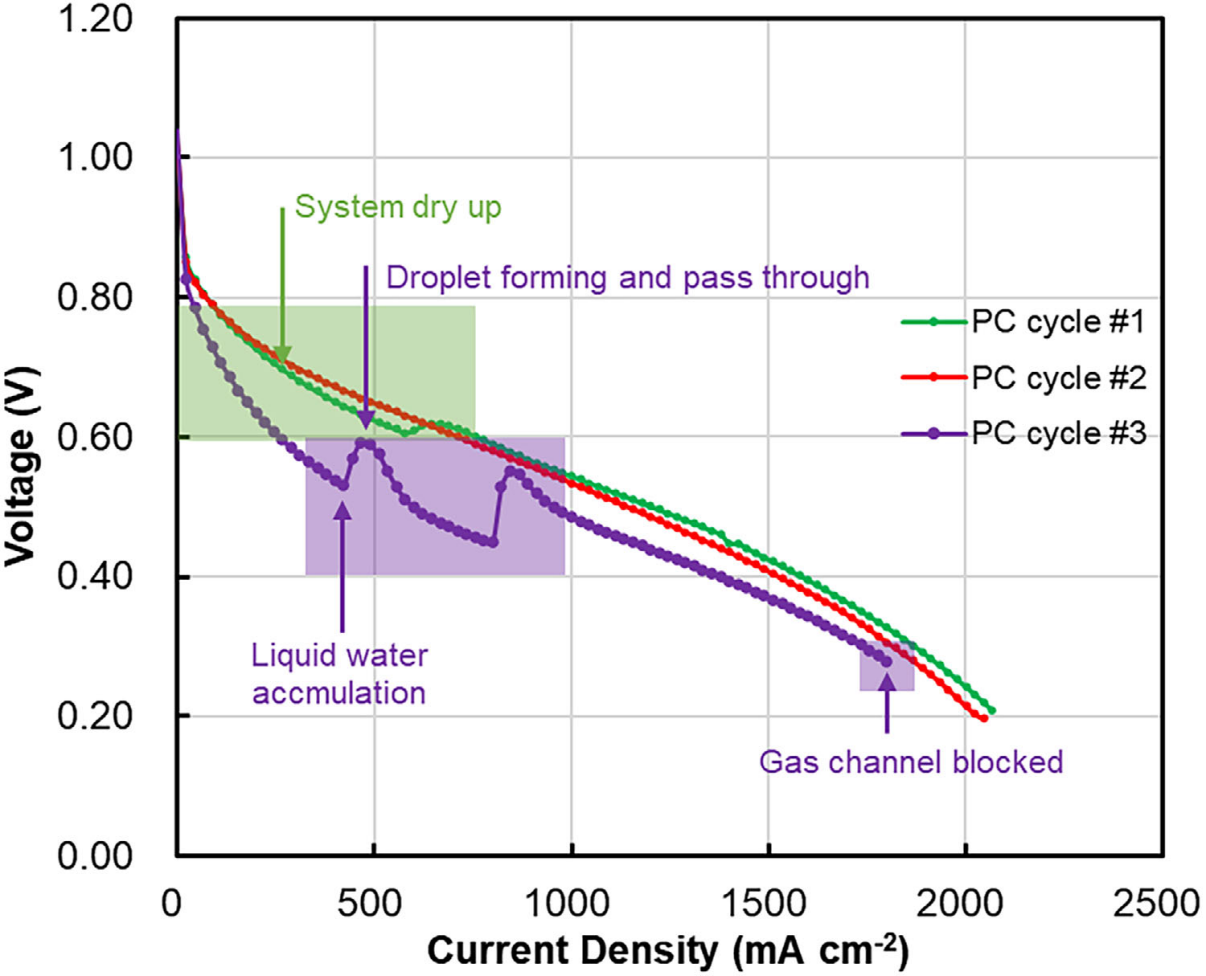
Schematic of fuel cell dry‐up and flooding behaviour during the polarization test. PC refers to the polarization curve. Three curves were recorded from 3 cycles of polarization test by using a cell temperature of 60 °C, a humidifier tank temperature of 70 °C, and external tube heating of 70 °C.

In LT‐PEMFC operations, flooding is a primary cause of system instability. This issue primarily arises from the accumulation of liquid‐phase water within the cell, which blocks the gas transport channels, leading to rapid consumption and depletion of reactants, and potentially causing a sharp decrease in current or even an open circuit. **Figure** **7** indicates the polarization curve response of the fuel cell system in both dry‐up and flooding conditions, where the cycle #2 curve represents the fuel cell response in normal conditions. In the dry‐up condition, activation losses increase at lower potential ranges (indicated by the green shading in Figure 7), resulting in a lower performance curve (cycle #1) compared to the normal condition (cycle #2). Dry conditions lead to inadequate hydration of the ionomer, reducing the number of three‐phase boundaries and thereby hampering efficient proton transfer. This limitation at the catalyst level restricts the system's reactivity and introduces greater activation resistance than usual. As voltage continues to decrease and current increases, moisture produced by the ORR adds some humidification to the system, which is why cycle #1 eventually shows similar performance to the normal system after an increase in current.

However, the water generated by the ORR is not always beneficial. After several test iterations, excessive water accumulates, leading to localized blockages of gas channels and active sites, thereby degrading performance in the low potential region (cycle #3) more than in dry conditions. With rising current, more water forms as humidification and reaction progress, causing liquid water droplets to form inside the electrode. These droplets are expelled from the fuel cell with the gas flow, leading to two jumps in cycle #3′s performance in the mid‐potential region. Ultimately, in the high current region, a significant amount of liquid water accumulates within the fuel cell and cannot be effectively drained, completely obstructing the gas channels. The electrode surface reactants are then consumed to the point of depletion, cutting off the current to the system. This results in an immediate disruption, as depicted in the image. In conclusion, minimizing liquid water within the fuel cell at a system level and achieving a balance between the rates of internal water accumulation and evaporation are critical factors in enhancing system stability and mitigating flooding.

### 2.5 Ideal Catalyst Layer Structure for Interlayer Interaction

**Figure 8:**
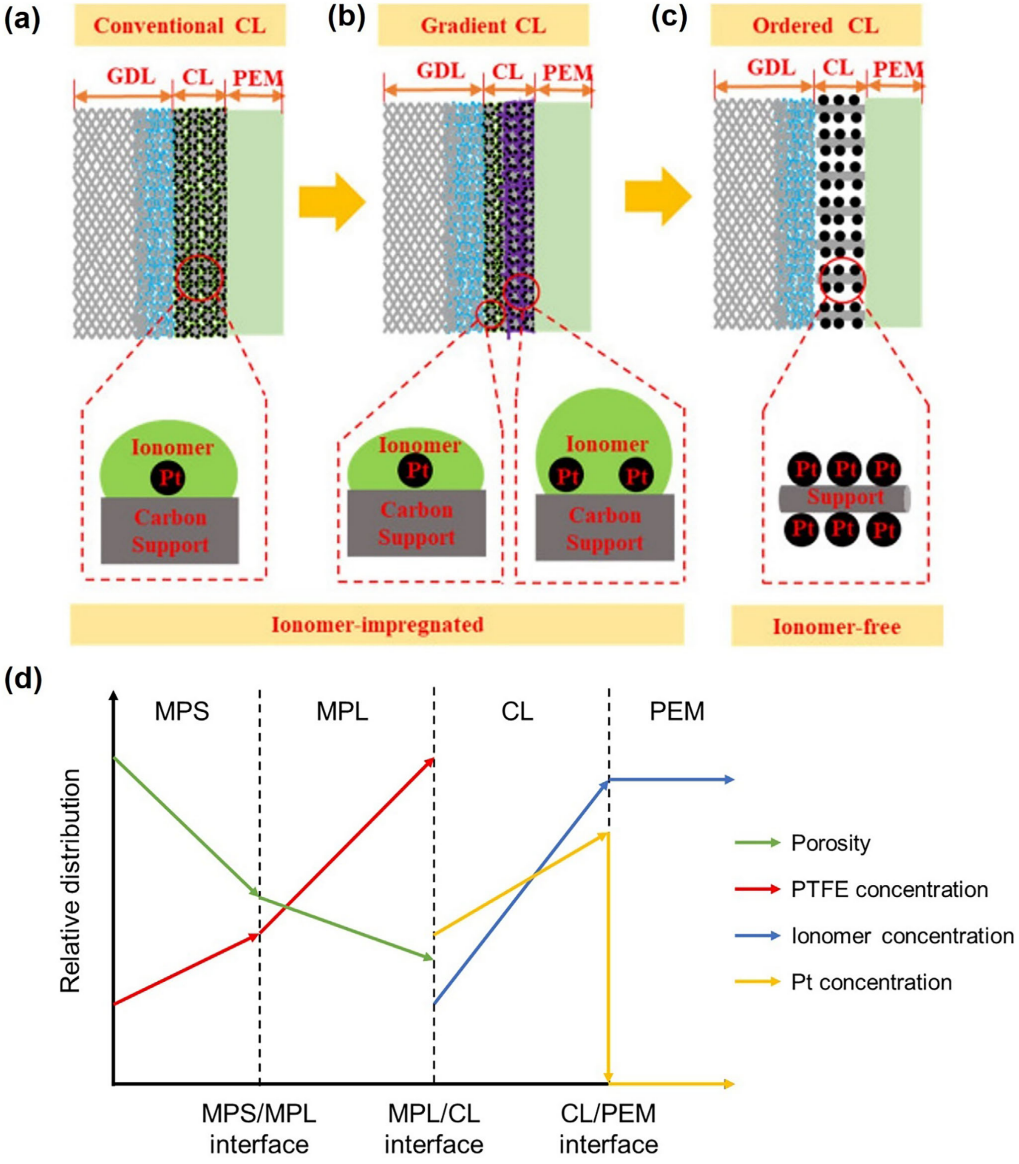
Schematic of the development progress of the membrane‐electrode‐assembly (MEA) structure. a) Conventional random deposition layer MEA. b) Gradient layer MEA. c) Novel macroscale order structure by using vertically aligned nanotubes to construct the catalyst layer. Reproduced with permission.^[^ ^110^ ^]^ Copyright 2020, Elsevier. d) Relative components distribution for porosity, Polytetrafluoroethylene (PTFE) concentration, ionomer concentration, and platinum particles concentration in gradient layer MEA schemes. While MPS/MPS refers to the interface between macroporous support (gas diffusion support) and microporous layer, MPL/CL refers to the interface between microporous layer and catalyst layer, and CL/PEM refers to the interface between catalyst layer and proton exchange membrane.

The MEA inhomogeneous gas diffusion and reaction‐coupled system presents the need for a graded design and ordered morphology. **Figure** **8** summarises the current development process of catalyst layer structures based on interlayer interactions.^[^ ^110^ ^]^ The conventional catalyst layer design (Figure 8a) has a uniform and disordered distribution between each layer, where different functionalities are attained through varied interlayer materials. This conventional approach often fails to achieve uniform performance distribution within a single functional layer due to the functional heterogeneity between layers. Gradient designs have been proposed to introduce concentration differences across individual functional layers (Figure 8b). These include gradients in platinum content, ionomer content, hydrophobicity in macroporous support, PTFE within microporous layer, porosity, and sulphonic acid content within proton exchange membrane.^[^ ^111^ ^]^ Ordered catalyst layer are constructed with a precisely arranged and ionomer‐free architecture, in which platinum nanoparticles are uniformly distributed on the support in a highly ordered manner (Figure 8c). Literature on gradient structures suggests an ideal concentration distribution, depicted schematically in Figure 8d. Although higher porosity in gas diffusion support (macroporous layer) reduces gas transport resistance, it also diminishes electrical conductivity, and large porosity at macroporous support/microporous layer interlayer contacts exacerbates contact resistance. Zhan et al. recommended that porosity in macroporous support should linearly decrease from the electric bipolar plate towards the macroporous support/microporous layer interface.^[^ ^112^ ^]^ Similarly, the microporous layer's porosity also linearly decreases from the macroporous support/microporous layer interface towards the catalyst layer side, while it should maintain consistent porosity at both interfaces to reduce contact resistance.^[^ ^113^ ^]^ Regarding hydrophobicity, the gradient concentration of PTFE manages the layer's hydrophobic properties. Xing et al. noted that the capillary effect is a primary mechanism for water removal in PEMFCs at lower reactant flow rates.^[^ ^114^ ^]^ Therefore, the capillary diffusion coefficient near the catalyst layer should be higher to enhance the removal of excess product water, with PTFE content higher near the catalyst layer and decreasing linearly toward the electric bipolar plate side (Figure 8d). Similarly, the PTFE concentration distribution in the microporous layer follows the same principles as on the macroporous support side. However, the content at the interface needs to be precisely controlled in order not to allow PTFE to impede gas transfer. The ionomer is the crucial proton‐conducting material in PEMFC, which must be sufficiently and evenly distributed to sustain the proton transport channel. Otherwise, excessive ionomer can cause gas transport to be blocked. For electrodes with 0.5, 0.25, and 0.1 mg cm^−2^ platinum loading, Sasikumar et al. concluded that the optimal Nafion ionomer loadings were 20%, 40%, and 50%, respectively.^[^ ^115^ ^]^ In gradient schemes, higher ionomer content should be closer to the proton exchange membrane side, while the microporous layer side should remain as clear as possible to facilitate gas transfer.^[^ ^116^ ^]^ Additionally, research has also focused on the optimal gradient distribution of platinum particles within the catalyst layer.^[^ ^117^, ^118^ ^]^ Although a higher platinum content on the gas diffusion layer side at high current densities helps establish a three‐phase boundary in humid conditions, it increases gas transfer resistance. The optimal platinum gradient should decrease from the proton exchange membrane side towards the microporous layer side.

**Figure 9:**
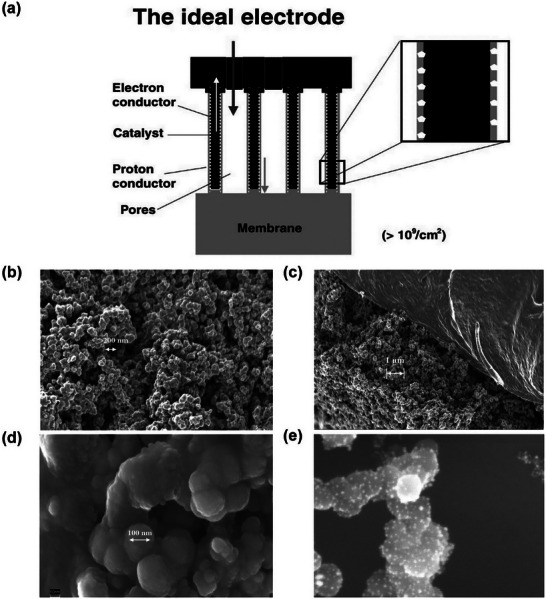
a) Schematic of ideal catalyst layer structure with the vertically aligned nanostructure between the proton exchange membrane and gas diffusion layer. b–e) High‐resolution scanning electron microscopy images of conventional randomly deposited catalyst layer structures. The white dots on (e) presented platinum particles loaded on a carbon support. Reproduced with permission.^[^ ^119^ ^]^ Copyright 2002, Elsevier.

Current trends in catalyst layer structure design focus on achieving well‐defined electron, proton, and reactant transport pathways through macroscopically ordered architectures, aiming to reduce platinum loading and enhance the stability of the three‐phase boundary. The ideal catalyst layer structure, initially proposed by Middleman et al. in 2002 (**Figure** **9**a),^[^ ^119^ ^]^ envisions electron and proton conductors perpendicular to the proton exchange membrane, aiming to simplify the interlayer structure and enhance transport efficiency. High‐resolution SEM images in Figure 9b–e reveals that platinum predominantly accumulates in regions with limited reactant diffusion and ionomer coverage due to the disordered structure, resulting in inefficient platinum utilization.^[^ ^119^ ^]^ Therefore, an optimal structure should evenly distribute platinum across electron, proton, and reactant transfer pathways to maximize its utilization. From the perspective of interlayer interactions, the vertically aligned structure integrates reaction, conductivity, and mass transfer functions, aligning with requirements for structural consistency and functional differentiation. Recent vertically align based schemes, particularly those incorporating CNT arrays, demonstrate intriguing water management properties. The superhydrophobic nature of CNTs, along with the hydrophilic transformation of loaded platinum particles, endows VACNT schemes with a unique “hydrophilic head” and “hydrophobic feet.”^[^ ^81^ ^]^ While VACNT offers promising properties for achieving desired structures, challenges in synthesis and morphological control have constrained its scalability beyond laboratory settings, a topic to be explored further in this work.

In conclusion, **Table** **2** presents a comparative summary of the attributes of conventional disordered structures, gradient structures, and the latest vertically aligned nanostructured MEA. Overall, the evolution of MEA structures is transitioning from disorder to order, with a focus on prioritizing interactions in the design of the next generation of MEA.

**Table 2:** Comparative summary of the attributes of disordered structures, gradient structures, and vertically aligned nanostructured membrane‐electrode‐assembly (MEA).

	Cost and difficulty of manufacture	Platinum utilization rate	Interlayer conductivity	Interlayer mass transfer	Performance and durability
Conventional Structure	Low cost and easy synthesis	Low platinum utilization and high platinum requirement	Bad conductivity due to bad contact	Bad mass transfer due to flooding	Mild performance and durability
Gradient structure	High‐cost and relatively complex synthesis	High platinum utilization and reduced platinum requirement	Bad conductivity due to an unmodified contact surface	Relatively good mass transfer due to the graded design has better water removal	Good performance and good durability
Vertically align nanostructure	Very high cost and limited in lab scale	Highest platinum utilization rate and lowest platinum requirement	Good conductivity due to low resistance along the vertical direction	Good mass transfer due to clear, separate transfer channel, and integrate water management	Best performance and relatively good durability compare with disorder design

## 3 Interlayer Interaction Supported by Modified Carbon Nanotube

The construction of an ordered morphology in the catalyst layer currently takes precedence when considering interlayer interactions, catalyst performance and durability in PEMFC. Despite CB's widespread use as a catalyst support for PEMFC, its drawbacks, such as poor stability, susceptibility to corrosion, and unfavorable microporous structure, have hindered effective performance. CB's particle properties do not lend themselves well to achieving an ordered nanostructure arrangement perpendicular to the proton exchange membrane. Among various carbon‐based catalyst morphologies, CNTs stand out for their high surface area, longitudinal low resistance properties, and stable sp^2^ electronic structure, making them ideal catalyst supports to replace CB. Essentially, a CNT is a convoluted form of a graphene sheet, available in single‐wall and multi‐wall types depending on the number of layers in the curl, with MWCNTs consisting of multiple coaxial layers of SWCNT.^[^ ^120^ ^]^ Both SWCNT and MWCNT can achieve asymmetrical electrical characteristics, effectively reducing resistance when aligned perpendicular to the proton exchange membrane.^[^ ^81^ ^]^ Through extensive research in the field, MWCNTs have been more widely adopted in PEMFCs than SWCNTs, not solely due to their superior electrical conductivity.^[^ ^14^, ^57^, ^121^ ^]^ In terms of mechanical stability, the more robust multi‐walled structure enhances durability during prolonged start‐up and shut‐down cycling.^[^ ^5^, ^122^, ^123^ ^]^ Furthermore, in the highly oxidative cathode environment of PEMFCs, the higher degree of graphitization in MWCNTs provides enhanced corrosion resistance.^[^ ^124^, ^125^ ^]^ Furthermore, after decades of development, the preparation of CNTs via processes like CVD or plasma‐enhanced CVD has become mature, enabling effective control of CNT macroscopic morphology.^[^ ^83^, ^120^ ^]^ CNT emerges as an ideal material for realizing the next generation of vertically align nanostructures. Considering the high‐temperature operation of the CVD process, several solutions for vertically align structure based on metal oxides and conducting polymers have been proposed in recent years.^[^ ^84^, ^94^ ^]^ This section of the review discusses disordered catalytic support schemes, represented by CNTs, and ordered catalytic support schemes, comparing them with other materials featuring vertically align nanostructure schemes. Building on previous ideals of materials involved in interlayer interactions, this work focuses on the impact of design and modification schemes on interlayer interactions, alongside the performance and durability of catalytic materials.

### 3.1 Randomly Deposited Carbon Nanotube in Fuel Cell Applications

Under fuel cell operating conditions, maintaining interlayer mechanical adhesion in prolonged high‐humidity and highly oxidative environments, efficient heat conduction, stable mass transfer structures, and electrical conductivity are central to achieving favorable interlayer interactions. Extensive research has demonstrated that CNTs exhibit markedly superior stability to other carbon morphologies under highly corrosive conditions. This not only confers more reliable interlayer mechanical adhesion but also provides distinct advantages for long‐term operation and stack scaling.^[^ ^5^, ^58^, ^126^ ^]^ Concurrently, the exceptional thermal conductivity of CNTs demonstrates their potential for large‐scale applications.^[^ ^127^, ^128^ ^]^ However, CNTs exhibit a trade‐off between interlayer mass transfer (water management) and interlayer conductivity: their excellent conductivity stems from intact wall structures and sp^2^ C═C bonds. Whilst these inert hydrophobic walls hinder catalyst loading and uniform dispersion on the surface, potentially leading to localized mass transfer limitations and performance degradation. In light of this, this section reviews various CNT surface modification strategies to evaluate how these modifications enhance interlayer interactions and optimize overall MEA performance. Recently reported CNT modification strategies include both covalent and non‐covalent approaches.^[^ ^57^ ^]^ The covalent modification effectively alters the physicochemical properties of CNTs by introducing chemical groups onto the graphene layer of CNTs, resulting in the formation of covalent bonds with carbon atoms.^[^ ^129^ ^]^ However, the non‐covalent modification methods for CNTs regulate their physicochemical properties without disrupting the graphene framework structure. These modifications preserve the intrinsic conductivity of CNTs while imparting new surface characteristics of functionalities.^[^ ^130^ ^]^ Both approaches modify surface characteristics of CNTs to enable homogeneous distribution of platinum catalysts on CNTs, positioning CNTs as highly promising support materials for PEMFC applications.

#### 3.1.1 Covalent Modification of Carbon Nanotube

The essence of covalent functionalization of CNTs is an oxidation‐based technique. The oxidation process, assisted by acids or oxidizing agents (e.g., HNO_3_, H_2_SO_4_, K_2_Cr_2_O_7_, glacial acetic acid, and H_2_O_2_), introduces oxygen‐containing groups (e.g., ‐COOH, ─OH, and ─C═O) to the CNT surface.^[^ ^131^ ^]^ CVD, as the main technique for CNTs production, will produce by‐products such as graphite particles, amorphous carbon, and metal catalyst residues. The posttreatment of strong acid to purify crude CNT has been widely used in CNT‐supported catalyst solutions.^[^ ^132^, ^133^, ^134^ ^]^ This oxidation‐based modification significantly improves the chemical reactivity, hydrophilicity, and dispersion of CNTs in solution.^[^ ^26^, ^135^ ^]^ Moreover, covalent functionalization of CNTs is crucial for optimizing their interlayer structure and enhancing platinum catalyst loading. The intrinsic chemical inertness of CNTs, with their smooth and stable graphene structure, limits effective interaction sites for platinum particles, leading to uneven catalyst dispersion. Covalent functionalization introduces active chemical groups such as carboxyl, hydroxyl, or carbonyl onto the CNT surface, creating anchor points for platinum nanoparticles. This improves catalyst dispersion, utilization, and performance in electrochemical applications, especially in PEMFCs. Additionally, from an interlayer perspective, covalent functionalization enhances interactions between CNT layers, improving their arrangement and facilitating more effective charge transfer paths. As a result, it boosts the overall electrochemical stability and conductivity of CNT‐based materials. Besides oxidation‐based covalent modification strategy, amination, fluorination, and addition reactions are also employed to introduce functional groups to tailor CNT properties.^[^ ^136^, ^137^ ^]^

**Figure 10:**
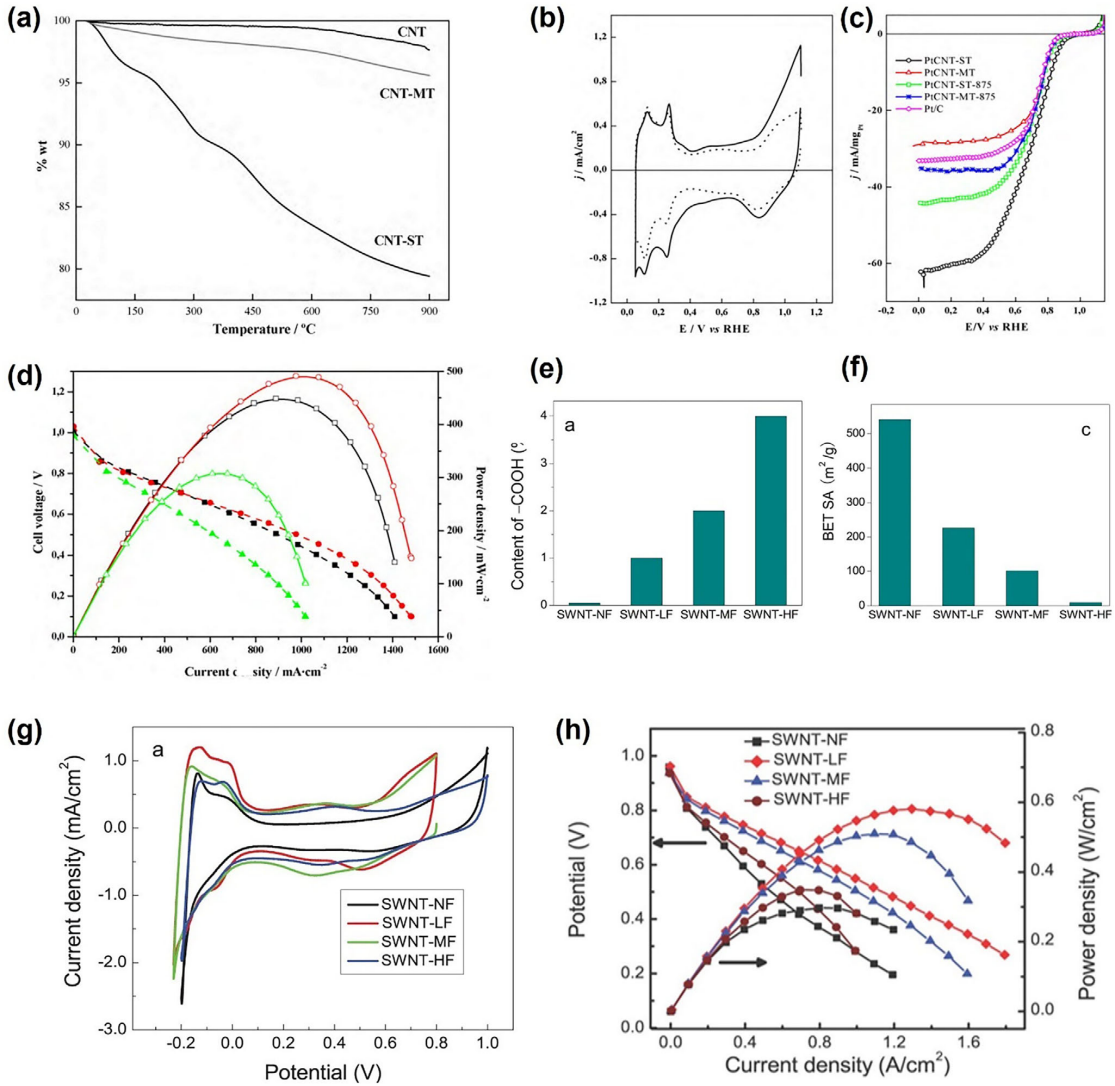
a) Thermogravimetric analysis (TGA) results of no acid treatment CNT, Mild acid treatment CNT (CNT‐MT), and severe acid treatment CNT (CNT‐ST). b) Cyclic voltammetry (CV) experiments result of Pt/CNT‐ST (solid line) and Pt/CNT‐MT (dashed line). c) Linear sweep voltammetry (LSV) results of the prepared catalyst compared with the commercial Pt/C catalyst. d) MEA polarization test of prepared Pt/CNT‐ST (red line) and Pt/CNT‐MT (green line) compared with the commercial Pt/C catalyst. Reproduced with permission.^[^ ^26^ ^]^ Copyright 2010, Elsevier. e) ─COOH group content for SWCNT with no functionalization (NF), low functionalization (LF), and high functionalization (HF). f) BET surface area of four prepared SWCNTs. g) CV results for Pt‐loaded SWCNT catalysts. h) Polarization test result for Pt‐loaded SWCNT catalysts. Reproduced with permission.^[^ ^40^ ^]^ Copyright 2013, Springer Nature.

However, varying oxidation intensities exert distinct effects on CNT structure and electrochemical performance. Moderate oxidation facilitates the formation of effective metal anchoring sites, whereas excessive oxidation may introduce structural defects that hinder electron transport. To further elucidate this balance, Hernández‐Fernández et al. systematically compared MWCNTs subjected to different acid treatment severities.^[^ ^26^ ^]^ They treated MWCNT with a mixture of H_2_SO_4_ (98%) and HNO_3_ (65%) and made a cross‐sectional comparison according to the severity of the treatment.^[^ ^26^ ^]^ Thermogravimetric analysis (TGA) implied that severely treated MWCNT (CNT‐ST) has a faster loss of mass with increasing temperature than mildly treated CNT (CNT‐MT) and untreated CNT (**Figure** **10**a), indicating more functional groups on the surface. Additionally, the Brunauer–Emmett–Teller (BET) surface area of CNT‐ST decreased by 55 and 84 m^2^ g^−1^ compared to regular CNT and CNT‐MT, respectively. The authors suggested that it is the surface functional groups that cause the surface pores to be blocked. In further electrochemical characterization, CNT‐ST exhibited a better ECSA than CNT‐MT (Figure 10b) and higher maximum current density (9 mA mg^−1^ higher at 0.7 V) (Figure 10c). Unfortunately, the performance improvement of the CNT‐MT catalyst over the Pt/C and mild acid‐treated catalyst was very limited (490 mW cm^−2^) compared with Pt/C and the CNT‐ST even has a worse maximum power density (310 mW cm^−2^) (Figure 10d).

Likewise, Jha et al. conducted an acid treatment on SWCNT.^[^ ^40^ ^]^ The highly functionalized CNT (SWCNT‐HF) group subjected to longer acid treatment time had more surface carboxyl content (Figure 10e) and a smaller BET surface area (Figure 10f). The detrimental effect of acid treatment on porosity was further confirmed by the decrease in BET surface area with increasing surface functional groups. In cyclic voltammetry (CV) tests, SWCNT‐HF with a high surface functional group content has the smallest ECSA. Conversely, the SWCNT‐LF group with the surface carboxyl groups removed by alkali had a larger ECSA compared to the pristine CNT, confirming the blocking of the surface carboxyl groups on the pore structure (Figure 10g). However, their work also highlighted that an increased presence of surface ‐COOH enhanced platinum particle dispersion and reduced particle size. The SWCNT‐LF sample also demonstrated the highest power density in the MEA polarization test (Figure 10h). In conclusion, acid treatment of CNT was beneficial in removing impurities from CNT to enhance porous structure, but excessive ‐COOH groups, while aiding in achieving better platinum dispersion and loading, had a suppressive effect on MEA performance. The presence of surface carboxyl groups increased the hydrophilicity of the catalyst layer, exacerbating water management issues and contributing to performance deterioration.

There is a trade‐off between improving the dispersion of CNT materials for PEMFC applications and maintaining their unique one‐dimensional morphology. Conventional chemical oxidation methods can activate the CNT wall and introduce polar functional groups, but intense chemical oxidation leads to truncation of the CNT and defects on the surface, which in turn disrupts the electron transport channel. Additionally, the chemical oxidation method causes more serious morphological changes in highly defective CNTs, making the optimal chemical oxidation parameters difficult to control and leading to variability in the quality of the treated CNT products. In recent years, the surface treatment of CNTs by plasma treatment has attracted the attention of researchers.^[^ ^138^, ^139^, ^140^, ^141^ ^]^ Plasma consists of ions, electrons, and neutrals, with a neutral overall charge, and can be divided into high‐temperature plasma and low‐temperature plasma.^[^ ^138^ ^]^ Plasma treatment technology allows for a variety of functionalization types, including the introduction of oxidative groups or nitrogen doping, by controlling the type of feed gas, varying the treatment power, and varying the treatment time.^[^ ^142^ ^]^

**Figure 11:**
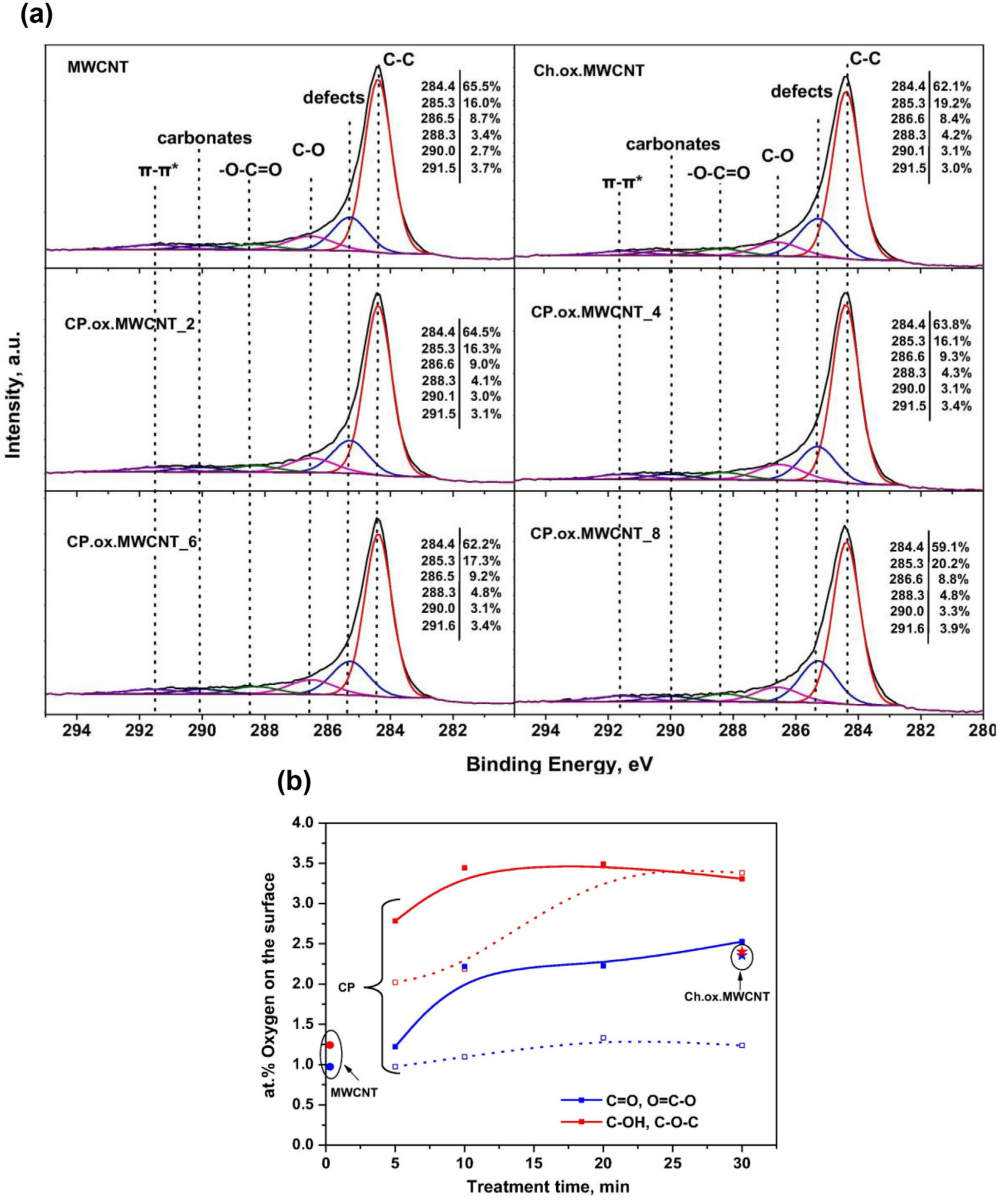
a) C 1s deconvoluted X‐ray photoelectron spectroscopy (XPS) spectra for the pristine MWCNT, oxidized MWCNT by chemical oxidation method, and cold plasma (CP) treated MWCNT, with treatment time varying from 2 to 8 h. b) O 1s deconvoluted XPS spectra of pristine MWCNT, oxidized MWCNT by chemical oxidation method, and cold plasma (CP) treated MWCNT. The solid line represents the sample treated with a higher plasma potential (26.2 V), and the dashed line represents the sample with a lower plasma potential (23.6 V). Reproduced with permission.^[^ ^143^ ^]^ Copyright 2021, Elsevier.

A typical scheme is the treatment of MWCNT with nanosecond pulsed dielectric barrier discharge (DBD) plasma under atmospheric pressure reported by Daletou et al.^[^ ^143^ ^]^ This functionalization can be achieved by high‐pressure nanosecond pulse (NSP)‐driven cold plasma discharge to introduce oxygen‐containing functional groups and defects on the MWCNT surface. **Figure** **11**a presents the result of the C 1s deconvoluted X‐ray photoelectron spectroscopy (XPS) spectra. In terms of defects (285.3 eV), cold plasma treatments of 2 to 6 h produce fewer defects than chemical treatments. The cold plasma treatment produced a higher surface carboxylic acid content (286.6 and 228.3 eV) than the chemical treatment. This showed that the treatment with plasma can introduce more surface polar groups while maintaining the MWCNT morphology. The O 1s deconvoluted XPS spectra results in Figure 11b revealed that there is a limit to the surface oxygen‐containing groups introduced using the cold plasma treatment, with a surface oxygen elemental content of about 6%. Higher treatment potential accelerated the saturation of the surface functional group. Similar adoption of cold plasma for SWCNT and MWCNT treatment was also reported by Mohan et al.^[^ ^144^ ^]^ Raman results indicated that after 20 min of cold plasma treatment, I_D_/I_G_ increased from 0.012 to 0.157 in SWCNT and from 0.895 to 1.22 in MWCNT. Furthermore, SWCNT after 20 min cold plasma treatment had a stronger ─OH stretch and ─C═O stretch than MWCNT in the FT‐IR results, which confirms the creation of more defects and polar groups on the surface of SWCNT. The reason for this phenomenon is that the limited penetration of cold plasma into the CNT makes the inner layers of the MWCNT subject to less cold plasma. In the subsequent ORR electrochemical tests, both SWCNT and MWCNT showed an increase in ORR activity after cold plasma treatment for less than 20 min, but more than 20 min of treatment time attenuated their ORR performance. The SWCNT after cold plasma treatment had higher ORR activity than MWCNT, which was attributed to the fact that the larger number of defects on SWCNT provided more oxygen adsorption sites.

**Figure 12:**
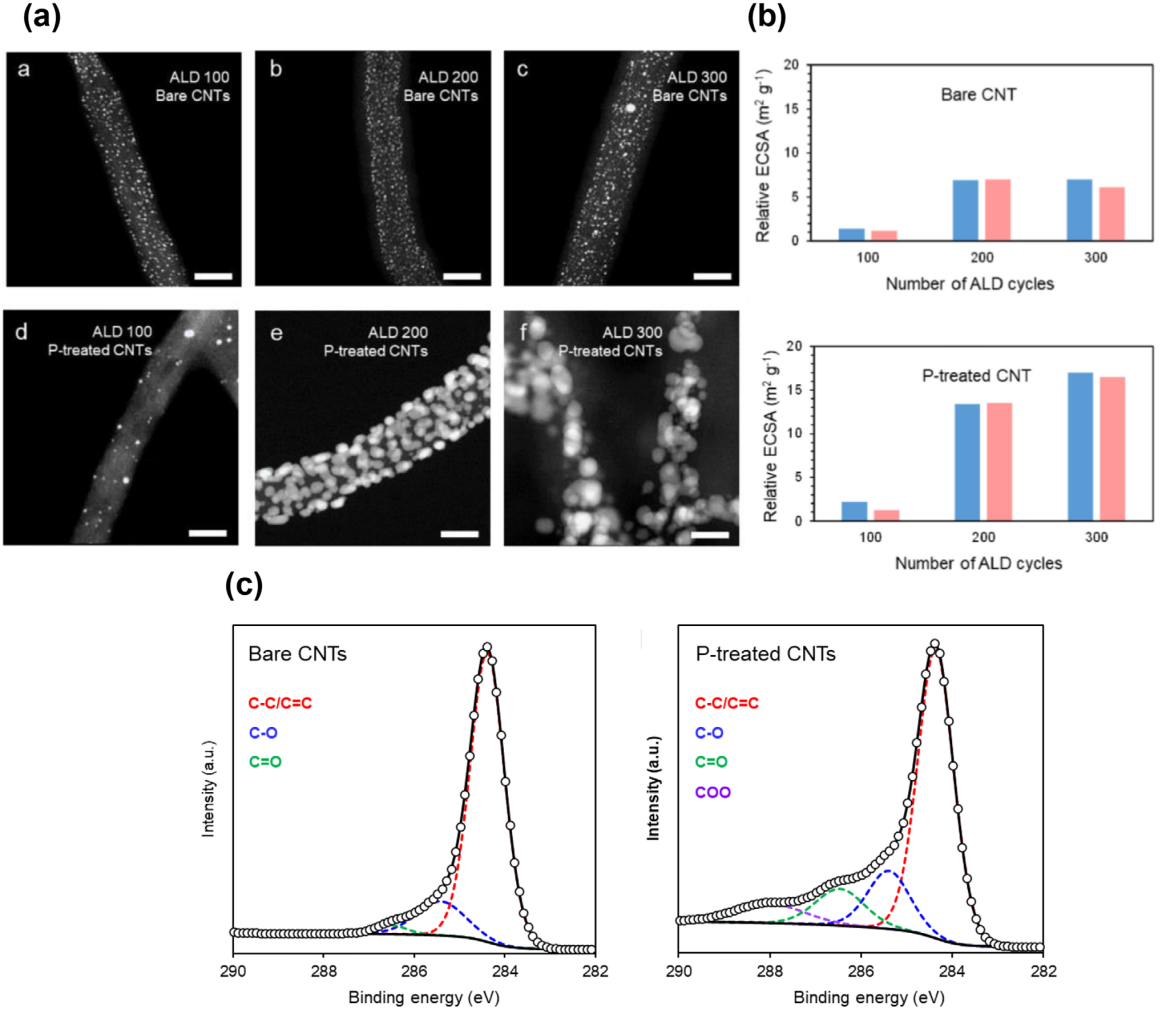
a) Scanning transmission electron microscopy (STEM) images of atomic layer deposition (ALD) of platinum particles on bare carbon nanotube (CNT) and plasma‐treated CNT with a number of ALD cycles of 100, 200, and 300. b) Comparison of ECSA for platinum loaded on bare CNT and plasma‐treated CNT before and after 5000 accelerated stress test cycles. c) C 1s X‐ray photoelectron spectroscopy (XPS) spectra for bare CNT sample and plasma‐treated CNT sample. Reproduced with permission.^[^ ^28^ ^]^ Copyright 2023, Elsevier.

The particle size distribution of platinum nanoparticles on plasma‐treated and untreated CNTs was investigated in a study by Koo et al.^[^ ^28^ ^]^ In their scheme, CNT was pretreated using O_2_ plasma at 90 W for 5 min. Subsequently, platinum nanoparticles were grown on the surface of CNT after several cycles using atomic layer deposition (ALD). The scanning transmission electron microscopy (STEM) image in **Figure** **12**a showed that the platinum particle distribution on the bare CNT samples remained dispersed as the number of ALD cycles increased, while the platinum particles on the plasma‐treated CNT surfaces presented an aggregated morphology. The surface platinum particle size of untreated CNT remained around 1.5 nm after 100 ALD cycles, and more ALD cycles did not significantly increase the size of platinum particles. However, the platinum particle size on the surface of plasma‐treated CNT showed a positive correlation with the increase in the number of cycles, with an average increase of 0.0248 nm per ALD cycle. Enhanced CNT surface energy after plasma treatment makes platinum in the ALD cycle tend to deposit on lower surface energy platinum particles, and Ostwald ripening increases the platinum particle size. The authors also performed ECSA tests (Figure 12b), and the plasma‐treated samples displayed greater ECSA and less decay after 5000 accelerated stress test cycles, which was attributed to the greater platinum loading causing higher ECSA and stability under long‐term operation. Finally, in the C 1s XPS spectra (Figure 12c), the plasma‐treated CNT presented an increase in the defect characteristic peak at 285.3 eV and the C‐O characteristic peak at 286.6 eV relative to the pristine CNT sample, confirming that the plasma treatment introduces surface oxygen‐containing functional groups and leads to the creation of structural defects.

Plasma pretreatment of CNTs can also achieve the purpose of introducing polar groups and creating surface defects to improve the platinum loading capacity of the CNT surface and the dispersion of CNTs in line with the chemical oxidation method. Compared to chemical oxidation, plasma treatment can achieve milder surface activation and retain the original structure of CNT to a greater extent by controlling the time an operation parameter, the treatment time is also greatly reduced. However, plasma treatment has disadvantages in terms of energy consumption and technological complexity. How to achieve the activation of CNT surfaces uniformly in a two‐phase gas‐solid reaction remains a difficulty in plasma treatment technology. Besides, for the multilayer structure of MWCNT, the plasma penetration is insufficient, so that the inner CNT walls are not fully activated, which also limits the application of this technique for the covalent modification of CNT support. High electrical energy consumption is also one of the impediments to the commercialization of the technology, with calculations by Daletou et al. showing that the energy consumption for cold plasma processing is around 200 g of MWCNT per kWh.^[^ ^143^ ^]^

Despite the obstacles and challenges that conventional chemical oxidation and emerging plasma surface treatment processes still face in achieving optimal surface oxidation of CNTs and enabling large‐scale engineering applications, the approach of introducing surface functional groups through covalent modification to enhance interlayer interactions in CNT catalyst remains promising. At the level of interlayer mechanical adhesion, acid treatment and plasma functionalization introduce polar functional groups onto the CNT surface. This enhances the affinity between the catalyst support and the polymer or Nafion membrane interface, thereby reducing interfacial delamination under operating conditions. Andersen et al. demonstrated through ^19^F nuclear magnetic resonance spectroscopy (^19^F‐NMR) that functionalization with nitric acid/sulfuric acid enhances Nafion adsorption onto CNT. The surface oxygen‐containing functional groups shift the adsorption kinetics from being governed by surface porosity to being controlled by surface oxygen groups.^[^ ^145^ ^]^ Experimental work has demonstrated that surface‐functionalized CNT additives with nitric acid reduce surface cracking.^[^ ^146^ ^]^ Youn et al.’s work confirmed that acid‐treated CNT sheets enhance the performance of flexible PEMFCs across all curvatures, attributed to the well‐preserved interlayer adhesion and contact maintained by the CNTs.^[^ ^147^ ^]^

Beyond mechanical adhesion, the strengthened interfacial bonding derived from covalent functionalization also facilitates more efficient energy transfer across the layers. Improved interlayers contact not only resists delamination but also provides a continuous pathway for heat transport, which directly influences the thermal management behaviour of the MEA. Owing to CNTs' inherent excellent thermal conductivity and improved interlayer adhesion, although functionalization has a minor effect on transverse thermal conductivity, surface functionalization also enhances interlayer heat conduction. Kaur et al. effectively reduced thermal interface resistance at both metal interfaces and CNT interfaces through surface covalent grafting.^[^ ^148^ ^]^ However, the extensive defects or wall discontinuities introduced by chemical oxidation processes disrupt local heat flux continuity, and engineering optimization based on operating conditions remains to be explored. Plasma modification, being a milder approach, can adjust surface properties while preserving the thermal conduction network, making it more suitable for thermal management requirements in large‐scale stacks.

Improved heat conduction ensures a more uniform temperature distribution across the MEA. However, the accompanying surface activation also alters the local hydrophilic‐hydrophobic balance. This transformation affects the interactions between water molecules and the ionomer at the interface. Furthermore, temperature‐induced variations in local water evaporation rates and catalytic activity lead to the redistribution of water within the interfacial region, which in turn influences mass transport under high current density conditions. Evidently, a strong coupling exists between thermal and mass transport phenomena within the MEA. Surface functionalization of CNTs primarily influences the affinity between the polymer and water. Molecular dynamics studies by Kikkawa et al. also indicate that the free energy distribution governing the permeation of oligomers across carbon material surfaces is influenced by the amphiphilicity of solvent molecules.^[^ ^149^ ^]^ Surface covalent modification enhances hydrophilicity, which improves the penetration depth of the polymer and reduces the free energy of surface water to stabilize the water‐carbon interface. This functionalization promotes the formation of continuous water films and proton channels, mitigating localized drying. However, it is crucial to note that excessive functional groups can clog pores, reduce hydrophobicity, and consequently cause localized flooding or increased mass transfer resistance. However, improving mass transfer through increased surface polarity often comes at the expense of electrical performance. The same functional groups that enhance wettability and proton conduction may disrupt the π‐electron network of CNTs, leading to higher electrical resistance and impairing electron transport. Therefore, a careful balance must be struck between catalyst dispersion and electron transport. Moderate modification preserves the conductive framework while providing anchor points for catalysts, achieving equilibrium between electrical conductivity and catalytic dispersion. Overall, the effects of covalent modification on interlayer interactions are interdependent; rather than isolated enhancements in one aspect, such as adhesion or wettability, can influence heat, mass, and charge transport simultaneously. Therefore, a holistic optimization of functionalization degree is essential to achieve balanced MEA performance.

#### 3.1.2 Non‐Covalent Modification of Carbon Nanotube

**Figure 13:**
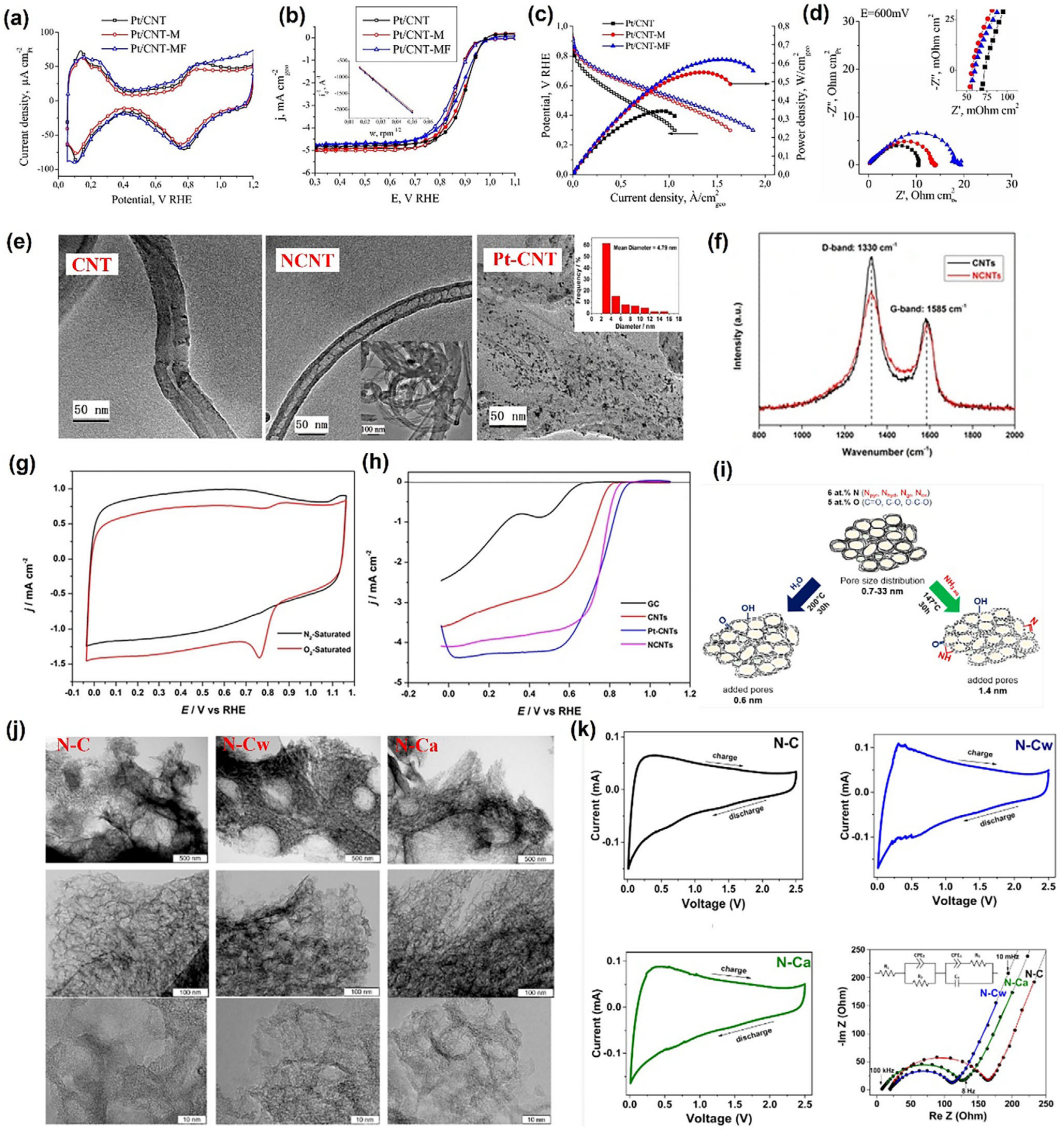
a) Cyclic voltammetry (CV) plot, b) Linear sweep voltammetry (LSV), c) polarization plot, and d) Nyquist plot for platinum loaded with untreated carbon nanotube (CNT), normal melamine–formaldehyde treated N‐doped CNT (NCNT), and melamine–formaldehyde resin treated N‐doped CNT. Reproduced with permission.^[^ ^27^ ^]^ Copyright 2019, Springer Nature. e) Transmission electron microscopy (TEM) images of CNTs, NCNTs and Pt/CNTs. f) Raman spectra of CNTs and NCNTs. g) CVs on NCNTs with a scan rate of 50 mV s^−1^. h) LSVs of bare glassy carbon, CNTs, Pt/CNTs and NCNTs modified glassy carbon electrode in O_2_ saturated 0.1 M KOH solution with a scan rate of 5 mV/s and electrode rotating rate of 1600 rpm.^[^ ^152^ ^]^ While NCNT refers to the N‐doped carbon nanotube. Reproduced with permission. Copyright 2015, Springer Nature. i) Schematic illustration of the structural evolution of N‐doped carbon sheets in the hydrothermal process after the treatment in water (N‐Cw) and aqueous ammonia solution (N‐Ca). j) TEM images of N‐doped carbon materials (N‐C) and after thermal treatment N‐Cw and N‐Ca. k) CV curves measured at a scan rate of 0.5 mV s^−1^ and EIS curves for N‐C, N‐Cw and N‐Ca. Reproduced with permission.^[^ ^153^ ^]^ Copyright 2020, MDPI.

For non‐covalent treatments, doping of heteroatoms such as N,^[^ ^27^ ^]^ S,^[^ ^150^ ^]^ and P^[^ ^151^ ^]^ has been reported for CNT tube wall modification. Among these, the pyridine structure formed by N‐doping on the carbon surface has received more attention due to the better increase of platinum anchor sites on the CNT surface. A variety of N‐doping reagents have been reported for N‐doped modified CNTs, such as melamine, mono‐ethanolamine, and ethylenediamine. Gribov et al. used melamine‐formaldehyde resin for the N‐doping modification of CNT, achieving a lower reaction temperature relative to conventional melamine doping.^[^ ^27^ ^]^ Follow‐up characterization demonstrated that the BET surface area of the CNT‐MF group using melamine‐formaldehyde resin for N‐doping was recorded as 160 m^2^ g^−1^, much lower than conventional CNT (260 m^2^ g^−1^) and conventional CB support (230 m^2^ g^−1^), but slightly better than conventional melamine‐treated CNT (130 m^2^ g^−1^).^[^ ^27^ ^]^ This suggests that N‐doping, akin to oxygen functionalization, also negatively affects the porous structure of the CNT. However, the TEM images still implied that the N‐doping gives the CNT surface platinum a narrower particle size distribution. The authors also performed CV, LSV and MEA performance tests on melamine‐formaldehyde resin‐treated N‐doped modified CNT. ECSA, mass activity, Tafel slope, and power density of N‐doped modified CNT were all recorded with better results (**Figure** **13**a–c). In terms of the electrochemical impedance spectroscopy (EIS) test, the two N‐doped CNTs had greater R_ct_ than the original CNTs, which indicates their poorer cathode performance (Figure 13d).

Nitrogen doping exhibits a complex dual effect: while pyridinic nitrogen sites improve metal dispersion and catalytic activity, excessive doping can introduce structural defects and hinder charge transfer. Consequently, different doping pathways and reaction environments may lead to distinct structural and electrochemical outcomes. To clarify these variations, several N‐doping strategies have been proposed and systematically investigated, such as plasma‐assisted N‐doping CNT,^[^ ^154^ ^]^ hydrothermal synthesis of N‐doping CNT,^[^ ^155^ ^]^ and furnace calcination‐treated N‐doping CNT.^[^ ^156^ ^]^ Tang et al. have introduced a microwave plasma‐synthesized N‐doped carbon nanotube (NCNT) to increase the ORR activity.^[^ ^152^ ^]^ The obtained NCNT exhibited a curly mass of tubular structures on the substrate, and its inside tube was divided into several conterminal compartments, which was different from the hollow inner tubes in pristine CNTs because of nitrogen substitution in the graphitic network, as shown in Figure 13e. The micrograph of the Pt/CNT catalyst synthesized with plasma reduction showed uniform dispersion of platinum nanoparticles on the CNT tube walls with an average platinum diameter of 4.67 nm. The Raman spectra in Figure 13f indicated a very slight increase in I_D_/I_G_ ratio (2.32 and 2.20 for NCNTs and CNTs, respectively), suggesting the surface imperfection of NCNTs and CNTs remained similar even after nitrogen doping. The CVs of NCNTs in O_2_‐saturated electrolyte in Figure 13g exhibited an intrinsic oxygen reduction process in the potential range from 0.04 to 0.85 V with a reduction peak at 0.76 V, suggesting a significant contribution to ORR activity. The LSV testing results in Figure 13h show that NCNTs exhibit superior ORR activity compared to bare GC and CNTs, with higher initial potential, half‐wave potential, and limiting current. This enhanced performance is attributed to nitrogen doping, which improves electron transfer and O_2_ adsorption on the CNT surface. In contrast to the plasma‐assisted synthesis, which relies on energetic species to induce nitrogen incorporation and structural rearrangement, hydrothermal treatment provides a milder and more scalable route for N‐doping, enabling simultaneous surface activation and pore engineering of CNTs. Okotrub et al. developed the hydrothermal synthesis of N‐doping CNTs under water (N‐Cw) and aqueous ammonia solution (N‐Ca).^[^ ^153^ ^]^ It turned out that the hydrothermal treatment caused both chemical modification and etching of the surface of carbon shells, resulting in a slight increase in specific surface area and volume of micropores and small mesopores, as illustrated in Figure 13i. TEM images in Figure 13j revealed that the original N‐C sample had a sponge‐like carbon structure with pores ranging from 5 to 30 nm, and the carbon shells consisted of corrugated, disordered graphene‐like layers less than 5 nm thick. After hydrothermal treatments, the N‐Cw and N‐Ca samples retained their sponge‐like morphology, but the graphene‐like layers became thinner and more defective due to etching, particularly at the edges. Owing to the increased specific surface area and pore volumes, the N‐Cw and N‐Ca exhibited superior electrochemical performance when used as electrode materials in electrochemical double‐layer capacitors and sodium‐ion batteries, as shown in the CV plots in Figure 13k. Specifically, the cathodic peak at 0‐0.2 V is related to the insertion of Na^+^ ions between the carbon layers, while the anodic peak at 0.3 V corresponds to the extraction process. The broad peaks at 0.6–0.8 V, which become more pronounced after hydrothermal activation, are likely associated with the adsorption of Na^+^ ions at atomic defects. The presence of these peaks suggests that the N‐Cw and N‐Ca possess good sodium storage capacity, particularly in regions with atomic defects, thereby enhancing the electrochemical performance of the batteries. Overall, the comparison between plasma‐assisted and hydrothermally treated N‐doped CNTs highlights that the choice of doping technique governs the balance between structural integrity, surface defect density, and electrochemical performance, underscoring the importance of tailoring the doping process to specific application demands.

Whether it involves strong acid treatment to augment the quantity of oxygen‐containing functional groups on the surface or enhancing platinum particle loading through N‐doping for CNT surface modification, the primary objective remains to increase the platinum particle loading capacity. There exists a trade‐off between enhancing active sites and maintaining an excellent conductive/mass transfer network: certain treatments reduce specific surface area/pore volume and may increase R_ct_, meaning not all non‐covalent strategies positively correlate with overall MEA performance. Different non‐covalent processes exhibit significant variations in activity enhancement, structural preservation, and process feasibility. Trade‐off selection and optimization must be guided by MEA engineering metrics (ECSA, R_ct_, polarization curves, long‐term accelerated stress test).

Beyond the macroscopic trade‐offs among surface activation, conductivity, and stability, it is essential to examine how non‐covalent modification at the molecular scale influences interlayer interactions within the MEA structure. Regarding interlayer interactions, non‐covalent doping can enhance interfacial adhesion with the polymer/ion‐exchange membrane by altering surface polarity or introducing surface defects. Molecular dynamics simulations confirm that coulombic interactions between the positively charged carbon surface resulting from N‐modification and the negatively charged sulfonate groups on the polymer side chains strengthen the attraction between the polymer and the carbon carrier surface.^[^ ^157^ ^]^ This surface modification enhances the effective coverage of the polymer on the carbon support surface under MEA operating conditions, thereby improving the MEA performance and platinum utilization.^[^ ^158^ ^]^ While interfacial adhesion governs the mechanical and electrochemical stability of the catalyst‐polymer interface, the modification‐induced changes in surface polarity and defect structure also exert a pronounced influence on water and gas transport across the catalyst layer. At the interlayer mass transfer level, although surface defects and N‐doping alter local hydrophilicity/hydrophobicity and pore structure, the resulting more uniform distribution of surface platinum particles actually contributes additional reactive sites, thereby enhancing fuel cell performance in high current density regions. Consequently, the optimal concentration and treatment duration for nitrogen source reagents must be determined, with subsequent studies exploring these optimal conditions. In addition to optimizing mass transport pathways, maintaining efficient charge and heat transfer within the CNT network is critical for sustaining high‐performance operation under dynamic load conditions. Regarding the interlayer electrical and thermal conductivity of CNT‐based catalysts, non‐covalent methods cause minimal structural disruption to the CNTs. This approach preserves the CNTs' longitudinal conductive structure to minimize electrical energy loss within the catalyst layer while retaining the CNTs' thermal conductivity advantages. This is beneficial for thermal management at the stack scale. Non‐covalent modification enables a fine balance between interfacial interactions and the preservation of intrinsic CNT properties, offering a promising strategy to harmonize mechanical adhesion, transport phenomena, and conductivity within advanced MEA architectures.

#### 3.1.3 Blended Carbon Black and Carbon Nanotube for Synergistic Effects

Moreover, in addition to the chemical modification of CNTs themselves, the synergistic effects arising from carbon‐based material combinations also contribute to enhancing the application of CNTs in PEMFC and improving interlayer interactions. Regarding the microporous layer, a substantial body of work has demonstrated that the combination of CNTs and CBs offers advantages over CNTs alone in constructing crack‐free micromesh structures,^[^ ^50^ ^]^ enhancing the water storage and drainage performance of MEAs,^[^ ^52^ ^]^ and reducing internal resistance within MEAs. As for the catalyst layer, the straightforward and widely used blending of CB with CNT proves to be an efficient strategy for improving interlayer contact between PEMFCs, enlarging the three‐phase interface, and generating synergistic effects. Currently, several studies have proposed the synergistic effects of CB and CNT blends.^[^ ^41^, ^159^, ^160^, ^161^, ^162^, ^163^, ^164^ ^]^ The primary advantage of blending CB and CNT lies in the tubular structure of CNTs, which connects the initially dispersed CB clusters, forming a highly conductive network structure. This structure inhibits the formation of surface cracks caused by the coffee‐ring effect during the drying process of conventional pure CB materials, thereby enhancing the surface smoothness of the catalyst layer. Moreover, CB serves as a more favorable site for platinum loading than CNT, improving the dispersion and platinum loading capacity of pure CNT materials and providing additional anchor points for the hydrothermal reduction process of platinum particles. Other reported benefits include superior electrical conductivity compared to pure CB materials,^[^ ^161^ ^]^ enhanced resistance to electrochemical oxidation,^[^ ^162^ ^]^ and increased porosity.^[^ ^163^, ^164^ ^]^ However, the current blending solutions of CB and MWCNT have limited applications in MEA. In some instances, MWCNTs were used as additives, directly blended with commercial Pt/C catalysts and tested for performance,^[^ ^163^ ^]^ while in other cases, a challenging sputtering process was employed to load a minimal amount of platinum on top of the blended material,^[^ ^160^ ^]^ which does not meet the requirements for commercialization studies.

**Figure 14:**
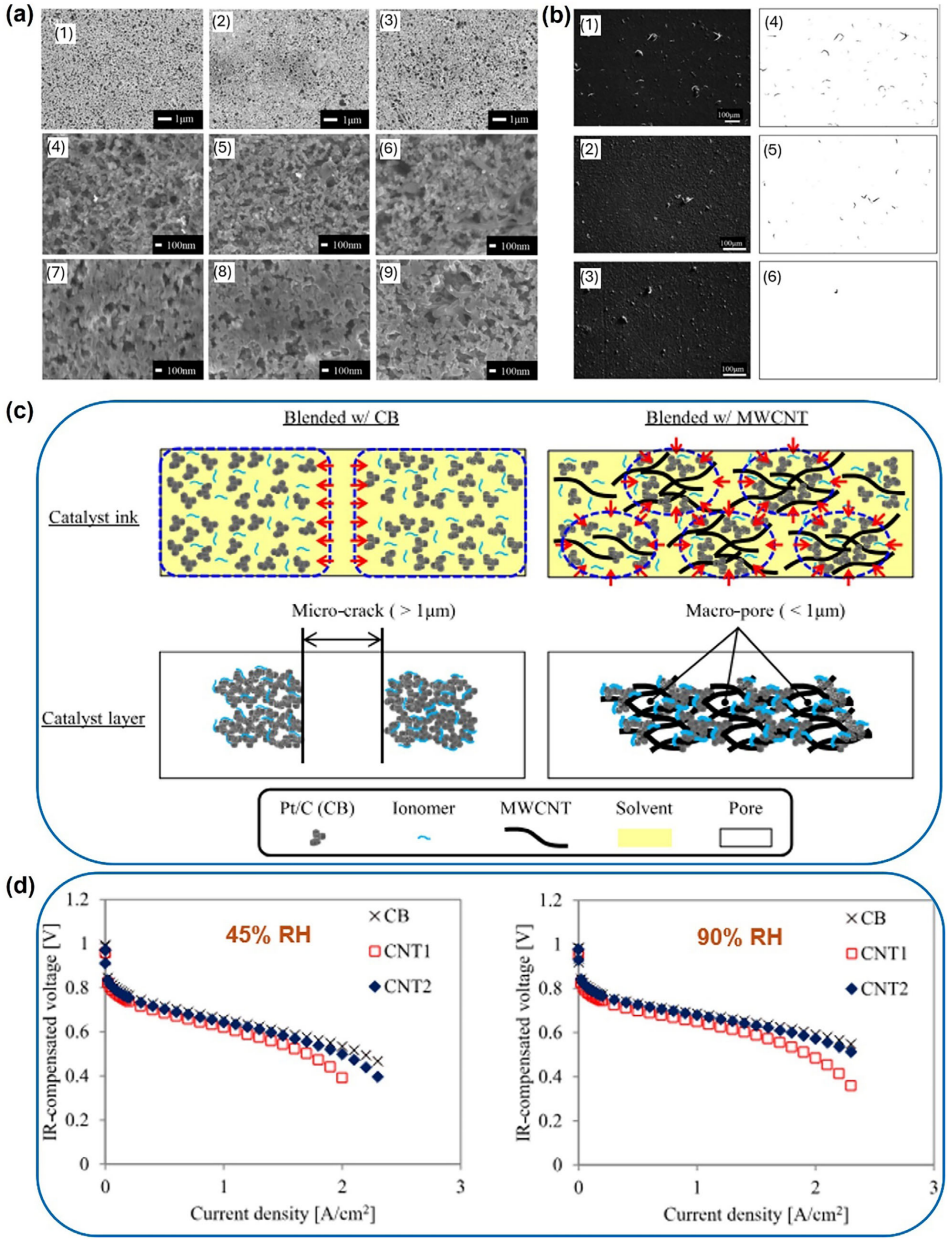
a) Scanning electron microscopy (SEM) images of platinum blended with carbon black (CB) (1, 4, 7), with small‐diameter carbon nanotube (CNT) (2, 5, 8) and big‐diameter CNT (3, 6, 9). The upper images (1, 2, 3) are in‐plane images at low magnification. The middle images (4, 5, 6) are in‐plane images at high magnification. The lower images (7, 8, 9) are through‐plane (CP‐treated) images. b) Microscope observation of the catalyst layers by irradiating transmitted light. (1), (2), and (3) are microscopic images of the surface on platinum blended with CB, small‐diameter CNT, and big CNT, respectively. (4), (5), and (6) are binary images of these. c) Schematic of Pt/C blended with MWCNT to construct a conductivity network and reduce the crack. d) Polarization curve of platinum blended with CB, with small‐diameter CNT and big‐diameter CNT. Reproduced with permission.^[^ ^163^ ^]^ Copyright 2015, Elsevier.

Suzuki et al.’s scheme was a typical example of how to investigate the porosity changes caused by carbon‐based additions by co‐blending commercial Pt/C catalysts with CB, short‐OD CNT, and long‐OD CNT. For simple mixing purposes, their work only employed centrifugal dispersion followed by ultrasonic mixing.^[^ ^163^ ^]^ **Figure** **14**a presents the SEM images of the three blends at different scales. At a relatively larger scale of observation (1 µm), the surface morphology of the three blends shows no significant differences. Microscopic images and cross‐sections reveal clusters of catalyst layer blended with CB, while in the material blended with CNT, tubular CNTs interspersed with CB clusters can be identified. CNTs with larger diameters exhibit more pronounced reticulation and tighter contacts. Additionally, the authors observed surface cracking on all three catalyst layers through a projection optical microscope. The addition of a larger diameter CNT resulted in a significant reduction in surface cracking relative to the conventional CB structure, as shown in Figure 14b. As illustrated in the schematic diagram in Figure 14c, the CNT acts as a linking bridge between the Pt/C clusters, reducing the tearing gap in the catalyst surface due to hydrodynamic action during catalyst layer drying. As previously discussed, fewer cracks and smoother surfaces are beneficial for improving interlayer contact and stability. Unfortunately, the electrochemical tests carried out in this work were very limited, and the results of the polarization test curves in Figure 14d for the three blended materials do not present a significant difference.

The advantage of CB and MWCNT is not only the formation of a tighter structure of the catalyst layer, but also the better material conductivity. The studies by Sumfleth et al.,^[^ ^159^ ^]^ Burmistrov et al.,^[^ ^161^ ^]^ and Ma et al.^[^ ^165^ ^]^ collectively highlight that the addition of CNT to the original pure CB reduces the percolation threshold of the material while enhancing its ductility and mechanical properties, underscoring the potential for using MWCNT and CB blends in PEMFC applications. However, the current research has predominantly focused on the linking effect and high electrical conductivity provided by MWCNT, overlooking other important properties such as the π‐electron interaction and catalytic performance enhancement of MWCNT as a catalyst support compared to platinum particles, as well as the radially low resistance of MWCNT after alignment. Additionally, the impact of the chaotic mesh structure on oxygen mass transfer resistance was not addressed. Therefore, further investigation is warranted into the co‐blending scheme of MWCNT and CB, aiming to enhance its potential as a catalyst carrier through moderate functionalization of MWCNT and microscopically ordered arrangement to increase the three‐phase interface and interfacial mass transfer.

From the perspective of interlayer interactions, the synergistic effects arising from blending carbon‐based materials such as CNTs and CB offer a straightforward and rapid approach to enhancing interlayer interactions. Regarding interlayer mechanical adhesion, CB's excellent dispersibility and film‐forming properties improve the uniform coverage of the catalytic layer, while the high‐strength framework of CNTs enhances overall structural stability. The blending of these two materials helps prevent cracking and delamination of the catalytic layer during operation, which can result from stress and water accumulation. Beyond improving mechanical adhesion, this synergistic framework also influences interlayer mass transport and water management, the microporous network provided by CB synergises with the hydrophobic channels of CNTs to form multi‐scale gas‐liquid pathways within the catalytic layer. This enhances reaction gas diffusion and liquid water drainage, thereby mitigating flooding or desiccation phenomena under dynamic load conditions. In addition to promoting efficient mass and water transport, the hybrid architecture further contributes to enhanced electrical connectivity, CNTs establish highly conductive long‐range electron transport pathways, while CB particles fill local voids and enhance point‐to‐point contact probability. They form a more continuous conductive network, reducing interfacial resistance between the catalytic layer and membrane. Finally, this structural integration also benefits interlayer heat transfer, although CB itself exhibits limited thermal conductivity, the incorporation of high‐thermal‐conductivity CNT frameworks improves local heat flux distribution. This assists the MEA in maintaining stable temperature gradients under high current densities.

### 3.2 Vertically Aligned Carbon Nanotube in Fuel Cell Applications

A vertically aligned structure is defined as one that is oriented along the z‐axis of a material, as opposed to being arranged in the x‐ or y‐plane. Such nano‐array structures exhibit distinct cross‐plane functional properties compared to those aligned along the x‐ and y‐axes.^[^ ^166^, ^167^, ^168^ ^]^ VACNT arrays have been utilized in the development of a wide range of sensor materials due to their unique morphology, low longitudinal resistance, superhydrophobicity, field effects, and mechanical flexibility.^[^ ^169^, ^170^ ^]^ Moreover, in the field of electrochemistry, the work of Qi et al. emphasises the specific advantages of vertically aligned structures in the development of supercapacitors.^[^ ^168^ ^]^ In his work, vertically aligned nano‐arrays were deposited directly on conductive substrates. This simplified the preparation process by avoiding the use of organic binders and conductive agents. These vertical arrays enhance the dispersion of active particles, improving both the quality of the particle loading and the electrochemically active surface area. The close contact between the vertical arrays and the substrate minimises contact resistance, leading to shorter conductive pathways and improved electron conductivity. Such properties of vertical arrays align well with the design requirements for fuel cell catalyst layers, offering a solution that closely approximates the ideal catalyst layer structure. In conventional catalyst layers with randomly distributed structures, the dispersion of ionomers on the catalyst surface is often associated with issues such as non‐uniform thickness, discrete distribution, and an unpredictable arrangement of the three‐phase interfacial structure. By contrast, the ordered structure of vertically aligned arrays is anticipated to improve ionomer distribution within the catalyst layer, providing uniform contact and enhancing the stability of the fuel cell system. However, unlike the randomly distributed structures, the ordered orientation of arrays, which is perpendicular to the material plane, needs to contend with gravitational and entropic effects during fabrication, presenting greater challenges to both the manufacturing process and the final properties of the material.^[^ ^166^ ^]^

To date, researchers have explored a wide variety of materials in the synthesis of vertically aligned electrodes, including metal oxides,^[^ ^84^, ^171^, ^172^, ^173^, ^174^, ^175^ ^]^ nanowires of platinum and its alloys,^[^ ^176^, ^177^ ^]^ conducting polymers,^[^ ^94^, ^178^, ^179^ ^]^ and CNTs.^[^ ^120^, ^169^, ^170^, ^180^, ^181^ ^]^ Due to excellent chemical inertness, metal oxide‐based vertical arrays exhibit promising durability for long‐term operation. For example, Zhang et al. utilized hydrogenated TiO_2_ nanotube arrays as a support structure, demonstrating a smoother ECSA loss profile after 18000 potentiostat scanning cycles compared to commercial Pt/C catalysts.^[^ ^84^ ^]^ However, impedance analysis under a fuel cell system revealed that this scheme had significantly higher resistance than conventional gas diffusion electrodes, which is attributed to the poor conductivity of TiO_2_ nanotubes. The work of Ozkan et al. pointed out that the conductivity of the TiO_2_ array catalyst layer is largely dependent on the choice of catalyst material. Despite this, the deposition of platinum in their study resulted in randomly distributed aggregates, which failed to establish a homogeneous conductive pathway, thus limiting the performance of metal oxide‐based arrays in fuel cells.^[^ ^173^ ^]^ To enhance the conductivity of TiO_2_ arrays, improvements such as annealing or carbon coating the outer layer of the nanotubes have been proposed.^[^ ^171^, ^172^ ^]^

Marconot et al. encountered additional challenges, such as insufficient catalyst‐specific surface area, limited effective catalytic sites, and water flooding in their tests of copper nanowire arrays in MEA, which ultimately led to suboptimal fuel cell performance.^[^ ^177^ ^]^ In contrast, Xia et al. constructed vertical arrays using a conducting polymer substrate. They employed a Nafion ionomer‐modified polypyrrole (PPy) substrate, where electrons were conducted through the polypyrrole matrix and protons were transported via the Nafion ionomer.^[^ ^94^ ^]^ Despite the innovative approach, the electrochemical in situ polymerization of PPy arrays on gas diffusion layer substrates did not result in the desired perpendicular alignment relative to the gas diffusion layer; instead, most PPy arrays were oriented perpendicular only to individual gas diffusion layer fibers. In summary, among the various substrates, MWCNTs have emerged as the most ideal candidate for synthesizing vertical array structures in PEMFC applications. This is due to their superior conductivity, large specific surface area, abundant mesoporous structure, potential for in situ growth, super‐hydrophobicity, and strong metal‐support interactions.

The unique properties of VACNT structures‐such as high longitudinal electrical conductivity, ordered arrangement, high specific surface area, and porous architecture—make them ideal materials for improving interlayer interactions. To enhance the overall performance of the MEA, optimizing interlayer interactions is crucial. The transport of electrons, protons, and oxygen at interfaces is essential for the formation of the three‐phase boundary and the efficient utilization of catalytic sites. This section first reviews the origins of VACNT performance in interfacial electron, proton, and oxygen transport to elucidate their influence on interlayer mass transfer and conductivity. Subsequently, it examines current VACNT synthesis methods and application bottlenecks within MEAs from an engineering perspective.

The vertical alignment of VACNTs relative to the proton exchange membrane direction maximises the radial electron transport capability of CNTs. However, the mechanical fragility of VACNTs, which makes them prone to breakage or entanglement under excessive bending, presents challenges for conductivity measurements. Conductivity testing methods, including two‐probe and four‐probe techniques, often require custom‐designed setups.^[^ ^182^, ^183^, ^184^ ^]^ These measurements are influenced by contact resistance and spatial resolution limitations, leading to reported conductivity values ranging from 200 to 10^5^ S m^−1^.^[^ ^185^ ^]^ A widely accepted conclusion is that VACNTs exhibit significantly higher electrical conductivity in the direction perpendicular to their growth plane than parallel to it. In a meta‐analysis by Bulmer et al., the exceptional conductivity of VACNTs was attributed to the intrinsic structural properties of CNTs, including ultralong mean free paths, high carrier mobility, and superior graphitic crystallinity.^[^ ^186^ ^]^ Moreover, the highly aligned VACNT arrays facilitate uniform tube‐to‐tube contact, reducing inter‐bundle tunnelling resistance compared to randomly oriented CNT networks. This enhances the radial electrical conductivity of VACNT structures. Souier et al. employed current‐sensing atomic force microscopy (CS‐AFM) with nanoscale resolution to characterize the cross‐plane electrical transport in VACNT arrays, further demonstrating their superior conductivity.^[^ ^187^ ^]^ The key factors contributing to this exceptional conductivity include the presence of continuous conductive pathways, high‐density packing that enhances carrier transport, and larger CNT diameters that reduce contact resistance. In conventional carbon‐based materials, the contact resistance at the interface between the catalyst layer and the gas diffusion layer or proton exchange membrane is often substantial due to the discrete nature of carbon particle contacts. In contrast, the highly ordered alignment of VACNTs enables the formation of continuous conductive networks. This direct connection reduces interfacial resistance associated with electron transport across multiple contact points, thereby improving interlayer electron transfer efficiency.

While optimizing interlayer electron transport, the VACNT array structure also presents great potential for improving interlayer proton transport. Efficient proton transfer between the catalyst layer and the proton exchange membrane is crucial for maintaining the stable operation of fuel cells. However, conventional catalyst support materials often suffer from discontinuous proton transport pathways and high interfacial resistance, which restrict proton diffusion and ultimately affect the overall performance of the fuel cell. In contrast, the porosity, tunable surface functionalization, and nanoscale array arrangement of VACNT structures provide new solutions for optimizing interlayer proton transport. Currently, the distribution of ionomers on catalyst surfaces and their interactions with catalysts have become key research focuses. Several advanced characterization techniques have been developed, including adhesion force mapping via atomic force microscopy (AFM),^[^ ^188^ ^]^ cryogenic transmission electron microscopy (Cryo‐TEM) for observing ionomer swelling,^[^ ^189^ ^]^ high‐angle annular dark‐field scanning transmission electron microscopy (HAADF‐STEM) combined with high‐resolution energy‐dispersive X‐ray spectroscopy (EDS) for elemental distribution analysis,^[^ ^190^ ^]^ compressed sensing reconstruction in scanning transmission X‐ray microscopy (STXM) using synchrotron radiation,^[^ ^191^ ^]^ small‐angle X‐ray scattering (SAXS) for investigating ionomer microphase separation,^[^ ^192^ ^]^ and small‐angle neutron scattering (SANS) for higher‐precision insights into ionomer–carbon interactions.^[^ ^193^ ^]^ However, most of these advanced characterization studies remain limited to conventional catalyst structures. Numerical simulations are still the primary method for understanding how VACNT structures improve interlayer proton transport by revealing ionomer distribution and proton conduction pathways.

**Figure 15:**
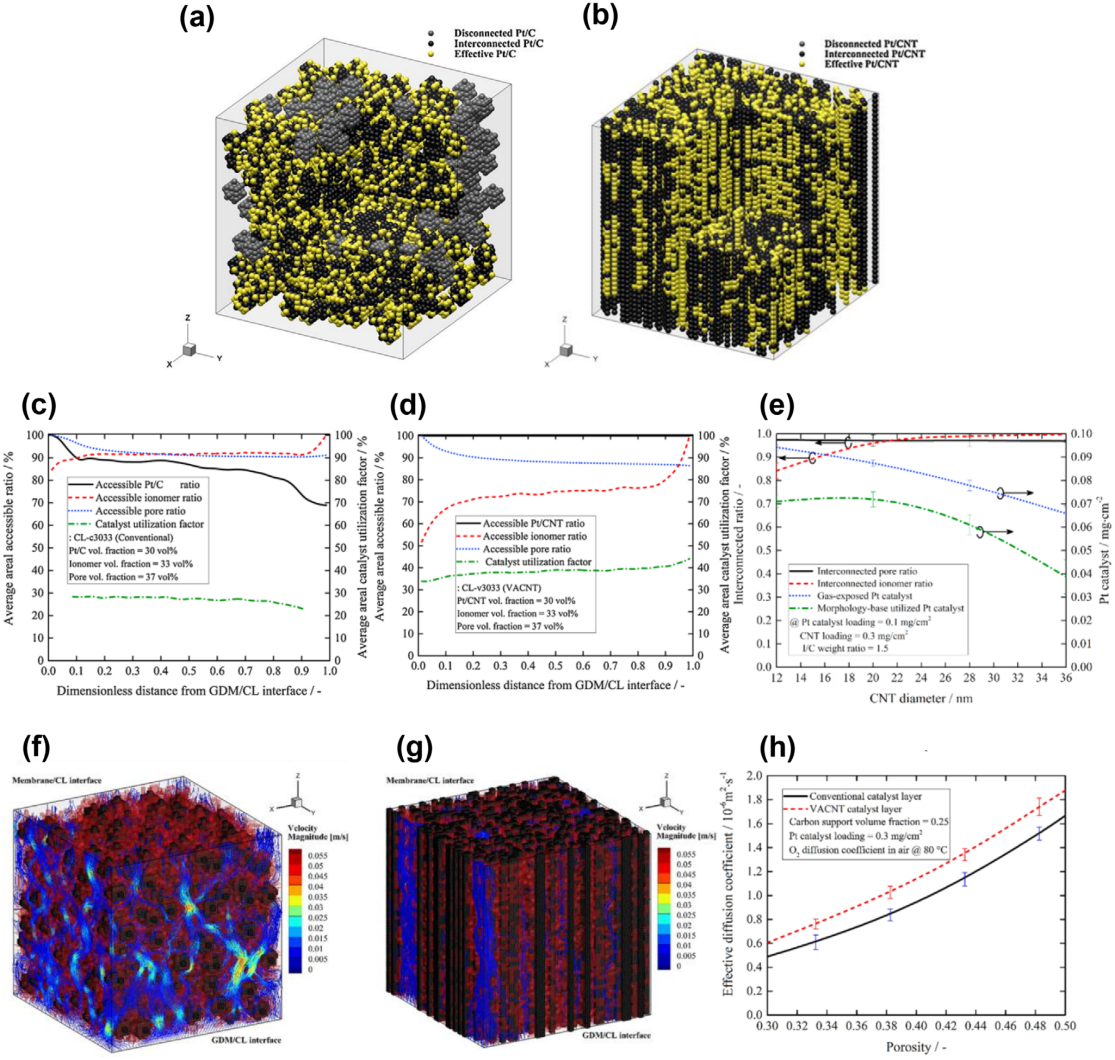
Schematic of 3D cutaway views of randomly generated effective catalyst layer simulation unit for a) Conventional Pt/C catalyst layer, and b) vertically aligned carbon nanotube (VACNT) array catalyst layer. The accessible ratio of catalyst particles, ionomer, pore, and catalyst utilization rate for c) conventional Pt/C catalyst layer, and d) VACNT array catalyst layer. Reproduced with permission.^[^ ^194^ ^]^ Copyright 2016, Elsevier. e) Effect of CNT diameter on interconnecting ionomer and porosity, gas‐exposed Pt catalysts site, and utilized catalysts in VACNT array catalyst layers at an ionomer‐to‐carbon (I/C) ratio of 1.5. Reproduced with permission.^[^ ^195^ ^]^ Copyright 2020, Elsevier. f–h) Schematic of velocity magnitude from gas diffusion layer/catalyst layer interface to proton exchange membrane/catalyst layer interface for (f) conventional Pt/C catalyst, and (g) VACNT array catalyst layer. (h) Comparison of simulated effective diffusion coefficient for conventional Pt/C catalyst layer and VACNT array catalyst layer varies with porosity. Reproduced with permission.^[^ ^196^ ^]^ Copyright 2019, Elsevier.

Shin et al. developed a quasi‐random nano‐structural modelling approach, using the Monte Carlo method to generate three‐dimensional quasi‐random nanostructures, simulating electron, ion, and mass transport pathways in VACNT‐based catalyst layers (**Figure** **15**a,b).^[^ ^194^ ^]^ Their findings indicated that catalyst accessibility was restricted by the aggregation of CB particles, with a calculated catalyst utilization of only 26.9%. In contrast, well‐aligned VACNT arrays achieved a catalyst utilization of 74.4%, representing a 24.7% improvement over randomly arranged CNTs (Figure 15c,d). Further studies highlighted that a CNT diameter of 20 to 30 nm and an I/C of 1.5 were optimal for catalyst utilization in VACNT structures (Figure 15e).^[^ ^195^ ^]^ Compared to randomly arranged CNTs and conventional Pt/C structures, the ordered inter‐tube spaces in VACNT arrays facilitated a more uniform ionomer distribution, resulting in clearer and straighter proton conduction pathways, thereby enhancing interlayer proton transport (Figure 15f,g). Numerical simulations based on the lattice Boltzmann method demonstrated that in VACNT arrays, 83.8% of the ionomer and 88.6% of the pores formed continuous transport pathways, significantly improving proton and oxygen accessibility (Figure 15h).^[^ ^196^ ^]^ However, despite numerical studies indicating that VACNT structures markedly enhance catalyst utilization and proton pathway connectivity, experimental validation remains limited. Future research should integrate in situ characterization techniques to gain deeper insights into ionomer distribution and dynamic behaviour within VACNT structures while assessing their long‐term stability and degradation mechanisms. This is essential to ensure the reliability of VACNT structures for practical fuel cell applications.

Under high current density operating conditions, oxygen transport resistance becomes a key limiting factor for PEMFC performance. As current density increases, effective oxygen diffusion within the catalyst layer becomes restricted, leading to insufficient oxygen supply to active catalytic sites. This reduces reaction efficiency and exacerbates concentration polarization.^[^ ^197^ ^]^ Furthermore, the random pore distribution and complex gas transport pathways in conventional catalyst layer structures further increase oxygen transport resistance, limiting its uniform distribution within the catalyst layer. Therefore, optimizing interlayer mass transport and reducing oxygen diffusion resistance are critical for enhancing overall fuel cell performance. Owing to their highly ordered array arrangement and tunable porosity, VACNT structures hold great promise for improving oxygen transport. Compared with conventional disordered CNT structures, VACNTs exhibit fewer inter‐tube entanglement points. Barrett–Joyner–Halenda (BJH) pore size distribution analysis has revealed that VACNTs primarily feature pore sizes of approximately 5 nm, which corresponds to the inner diameter of individual CNTs within the array.^[^ ^198^ ^]^ However, when VACNT arrays are grown on different substrate materials, increased tube curvature and entanglement introduce additional inter‐tube contact points that act as gas adsorption sites, leading to the appearance of mesoporous structures larger than 10 nm in the BJH pore size distribution.^[^ ^199^ ^]^ Babu et al. employed a high‐temperature CO_2_ treatment process to open the closed ends of VACNT arrays, thereby increasing the accessibility of internal pore structures.^[^ ^200^ ^]^ This treatment enhanced the BET surface area from 570 to 912 m^2^ g^−1^, while the total pore volume increased from 1.02 to 1.38 cm^3^ g^−1^. The formation of additional through‐pore structures benefits the highly ordered VACNT arrays, which typically suffer from a lack of externally accessible gas adsorption sites, thereby improving oxygen adsorption capacity. This further reduces oxygen mass transfer resistance at the catalyst layer interface, which is a crucial factor determining PEMFC performance under high current densities. Beyond optimizing oxygen transport, the unique surface characteristics of VACNTs also play a significant role in fuel cell water management. Their highly oriented alignment, combined with nanoscale roughness, imparts superhydrophobic properties that facilitate the regulation of water generation and removal. This, in turn, improves the reaction environment within the catalyst layer and enhances the overall stability of the fuel cell.

In terms of water management, the surface of VACNT arrays with nanoscale roughness has been shown to have superhydrophobic properties, and initial water contact angles of VACNT arrays have been reported to be between 126° and 156°.^[^ ^201^, ^202^, ^203^, ^204^ ^]^ Wirth et al. also observed the residence time of water droplets on the surface of VACNT arrays and the change in contact angle during contact angle tests. As the water droplets evaporated, the VACNT arrays gradually buckled and ultimately collapsed due to the pressure difference caused by the evaporating water, and the contact angle was transformed to a relatively hydrophilic 85°.^[^ ^203^ ^]^ The presence of air in the slits of the initial VACNT arrays, together with the VACNT arrays, forms a low‐energy surface, and capillary force‐driven water can penetrate the slits to form solid‐liquid contacts.^[^ ^203^ ^]^ Obviously, the inherent superhydrophobic properties of the surface and internal capillary action are favorable for fuel cell water management. These characteristics help mitigate the rise in reactant mass transfer resistance typically caused by water flooding, thereby improving overall cell performance. Meng et al. reported a scheme involving VACNT implementation for the catalyst layer and microporous layer bifunctional layer integration.^[^ ^29^ ^]^ It was demonstrated that the microporous layer‐free VACNT structure could still achieve effective water management and gas transfer functions. Additionally, they introduced metallic Co on the Pt side to enhance the hydrophilicity of the proton exchange membrane side through surface Co oxides. The results indicated that the contact angle on the Pt/Co side decreased from 128° to less than 90° in the absence of Co. The unilateral distribution of Pt/Co particles atop VACNT was attributed to the limited permeability of platinum particles in the magnetron sputtering method. They further emphasized that the optimization resulting from microporous layer removal not only led to a thinner gas diffusion layer but also enhanced convective mass transfer through the open flow field, thereby reducing mass transfer losses.^[^ ^29^ ^]^ MEA performance tests revealed an increase in current density from 1.5 to 2.13 A cm^−2^ for samples without a microporous layer compared to those with a microporous layer. In addition, the metal nanoparticles and Nafion distributed at the top of VACNT provided a certain degree of hydrophilicity when VACNT was used as a cathode, and the lower end of VACNT without metal nanoparticles remained highly hydrophobic. These properties facilitated the transport of H_2_O and the formation of H_3_O^+^. The absence of a PTFE‐treated microporous layer further enhanced the conductivity between the catalyst layer and macroporous support.^[^ ^29^ ^]^ Liang et al. developed a model of water flooding conditions in PEMFCs with VACNT arrays. In their model, under water flooding conditions, all gaps between the VACNTs were filled with water. Oxygen and proton conduction on the catalyst surface occurred through a thin Nafion layer coating the surface of the CNTs.^[^ ^205^ ^]^ Notably, their model gave specific optimal results for the morphology of the VACNT when constructed by the researcher, with an optimal spacing of 26 nm and an optimal length of 300 nm for the VACNT. The optimal thickness of the Nafion layer covering the surface of the VACNT is 1.7 nm. The thickness of the platinum‐Nafion subdomain is the main limiting factor in achieving the optimal VACNT array structure. In essence, having the appropriate thickness of the platinum‐Nafion subdomain is the key to achieving an effective three‐phase boundary in the VACNT array structure. Further modelling should consider the permeability and dispersion of the metal nanoparticles upon loading, and similar experimental work has been reported on the lack of permeability of metal nanoparticles, resulting in a majority concentration at one end of the VACNT.^[^ ^81^, ^83^ ^]^ The exceptional hydrophobicity and conductivity of VACNT arrays stem from the optimized voids and their perpendicular orientation relative to the proton exchange membrane. This configuration provides shorter mass transfer channels and conductive pathways, which are critical for meeting the operational demands of MEA. However, the vertical array structure also introduces significant challenges to the synthesis process.

Overall, the VACNT array significantly reduces mass transport losses and maintains stable water management performance under conditions of high current density (>1.5 A cm^−2^), moderate to high humidity (>70% RH), and frequent load switching. However, its advantages are less pronounced under low humidity or prolonged low‐load operation, with some results even approaching or falling below those of CB or graphene‐based catalytic layers. However, considering typical technical scenarios for fuel cells in heavy‐duty trucks, maintaining stable high‐current performance and durability under changing loads remains in line with market expectations. The application of VACNTs necessitates balancing the long‐term performance benefits from reduced platinum consumption against the fabrication costs associated with high‐temperature CVD growth and transfer processes. Variations in VACNT fabrication processes and structural evolution under operating conditions result in deviations from the ideal structure, leading to partial interlayer interaction functionalities falling short of expectations. Consequently, it is necessary to review VACNT solutions against the specific operating conditions of the MEA to assess their capability in achieving the desired interlayer interactions.

**Figure 16:**
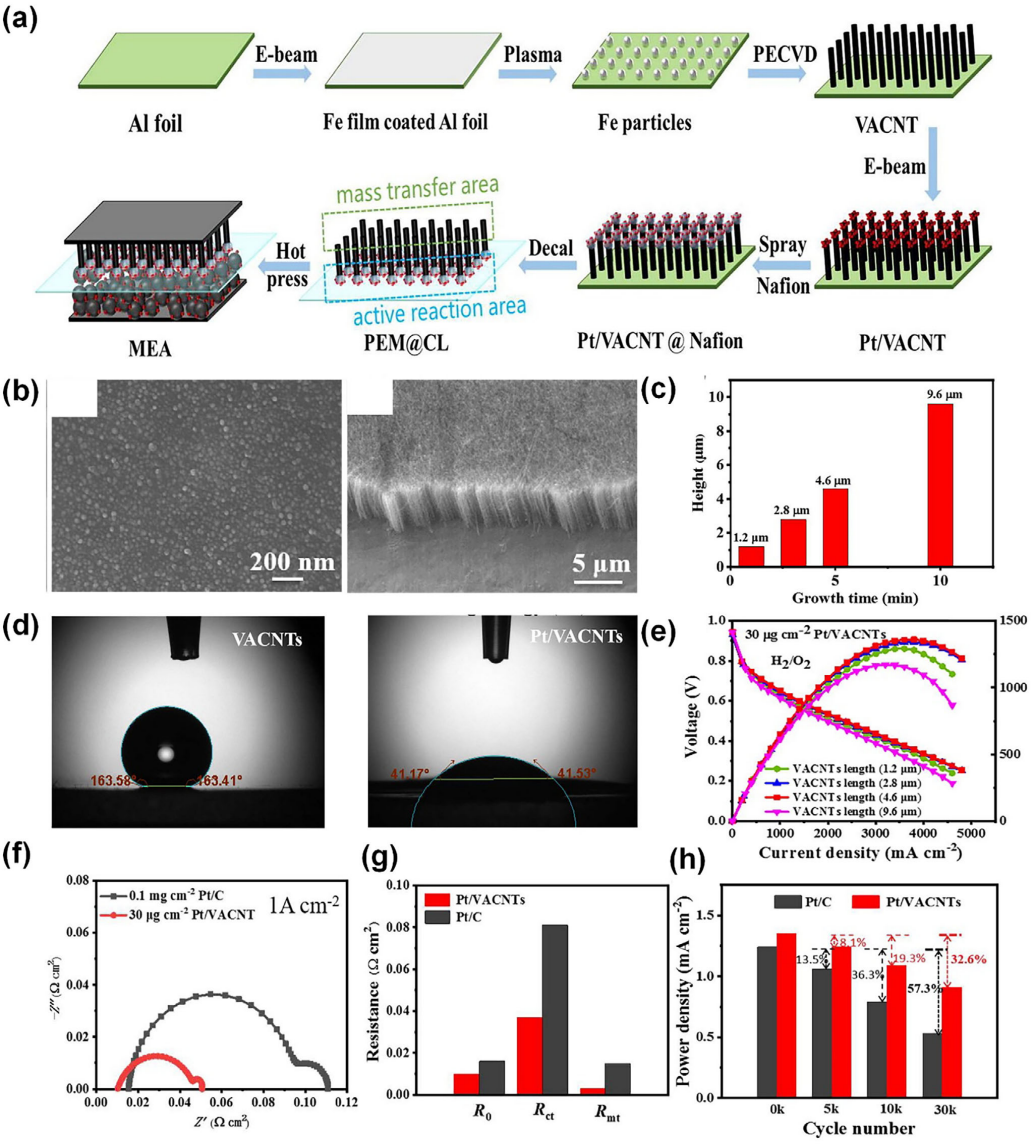
a) Schematic of platinum loaded on vertically align carbon nanotube (Pt/VACNT) catalyst preparation process. b) Scanning electron microscopy (SEM) images for the top view of VACNT and the cross‐sectional view. c) The length of the VACNT varies with growing time. d) Surface contact angle of VACNT and Pt/VACNT. e) Polarization of Pt/VACNT with different lengths. f,g) Nyquist plot and calculated resistance for Pt/VACNT sample with 30 µg cm^−2^ platinum loading. h) Decrease in the power density of Pt/VACNT compared with the conventional Pt/C catalyst. Reproduced with permission.^[^ ^83^ ^]^ Copyright 2022, Elsevier.

Chemical vapour deposition (CVD) is presently the most effective method to achieve in situ vertically aligned structures. The morphology of the resulting VACNT arrays can be precisely controlled by manipulating the deposition of the initial catalyst on the template and adjusting the duration of CVD.^[^ ^120^, ^206^ ^]^ In addition, factors such as gas diffusion of hydrocarbon feedstock, mechanical interactions between neighbouring CNTs, and catalyst feedstock also have an influence on the final VACNT array structure.^[^ ^207^ ^]^ Template‐based synthesis of VACNT arrays has been extensively validated, but the complete transfer of VACNT from templates to MEA remains a challenge.^[^ ^208^, ^209^, ^210^, ^211^ ^]^ The uniform loading of catalytically active metal nanoparticles and Nafion ionomers on VACNT, as well as the suitability of VACNT for wet modification processes, has limited its application in fuel cells. Meng et al. have proposed a scheme for growing VACNT on the surface of aluminium foil and transferring it to the proton exchange membrane surface for the preparation of MEA by the CCM‐DT method.^[^ ^83^ ^]^ They first loaded Fe particles required for CNT growth onto the surface of the aluminium foil by plasma treatment. The CNT was subsequently grown by an inductively coupled radio frequency plasma‐enhanced chemical vapour deposition (RF‐PECVD) system. The same e‐beam method as Fe particle loading was also used to load platinum particles onto the surface of VACNT (**Figure** **16**a). The SEM images in Figure 16b implied that the VACNT has a very neat vertical structure at the macroscopic level. Figure 16c revealed the VACNT length corresponding to different CVD treatment times. Similar to the weak platinum loading on the CNT wall, VACNT structures have also been reported to have difficulty loading platinum, with vapour phase deposited platinum only being distributed on top of the VACNT.^[^ ^81^ ^]^ The surface contact angle in Figure 16d demonstrated the hydrophobic differences in such an inhomogeneous structure. VACNT on the bottom has less platinum loading and retains its original super hydrophobicity with a water contact angle of 163.5°, much higher than the surface of conventional CB (136°). The top contact angle with more platinum loading was only 41.5°, proving to be hydrophilic. VACNT achieved hydrophilic and hydrophobic coupling in a single structure. With increasing humidity (from RH 30% to 100%), the output power increased from 0.43 to 0.79 W cm^−2^ (Figure 16e), indicating better water management within the MEA. Obviously, due to the poor platinum permeability, different lengths of VACNT will result in different ratios of hydrophobic and hydrophilic zones. Electrochemical test results presented that the 4.6 µm VACNT sample achieved a maximum power density of 1.36 W cm^−2^, which was a 17% improvement over commercial Pt/C (1.24 W cm^−2^). In addition, Pt/VACNT electrodes have a low R_ct_ (0.037 Ohm cm^−2^) and mass transfer resistance (0.003 Ohm cm^−2^) (Figure 16f,g). The durability test indicated that the Pt/VACNT only decreased by 32.6% in power density after 30 000 accelerated stress test cycles, but a 57.3% decrease in Pt/C (Figure 16h). The presence of shorter, clearer mass transfer channels enabled VACNT to form a more stable three‐phase boundary for the reaction.

**Figure 17:**
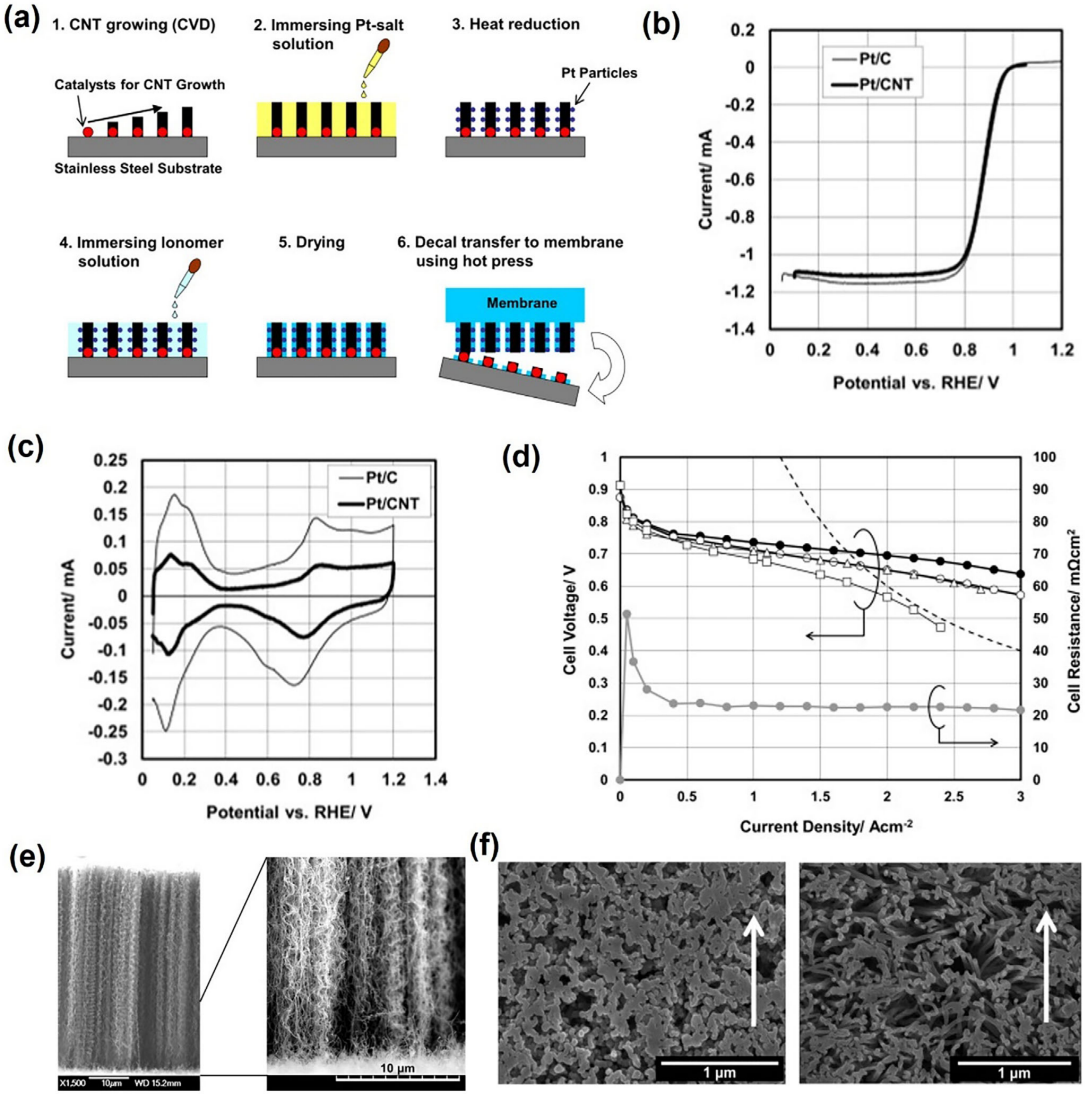
a) Schematic of the preparation process of the proposed platinum‐loaded vertically aligned carbon nanotube (Pt/VACNT) catalyst. b) Linear sweep voltammetry (LSV) plot of the Pt/VACNT scheme compared with the conventional Pt/C catalyst. c) Cyclic voltammetry (CV) plot of the Pt/VACNT scheme compared with the conventional Pt/C catalyst. d) Current‐voltage test performance test of Pt/VACNT electrode compared with conventional Pt/C electrode.°: VACNT electrodes (20 cm^2^ cell), ●: VACNT electrodes IR corrected (20 cm^2^ cell), Δ: VACNT electrodes (236 cm^2^ cell), □: conventional electrodes (236 cm^2^ cell), ●: cell resistance of VACNT electrodes. Dashed line: DOE target 0.125 g_PGM_ kW^−1^. e) Scanning electron microscopy (SEM) images of wavy VACNT. f) Cross‐sectional view of Pt/VACNT electrodes. Reproduced with permission.^[^ ^181^ ^]^ Copyright 2014, Elsevier.

From the perspective of interlayer interactions, the contribution of VACNTs to interlayer mass transfer primarily stems from their inherent hydrophobicity and shorter mass transfer pathways, enabling gases at the gas diffusion layer/catalyst layer interface to reach reactive sites more rapidly. Effective interlayer conductivity also relies on CNT structures oriented perpendicular to the proton exchange membrane to achieve minimal axial resistance. Maintaining consistent CNT spacing and vertical alignment within the array is essential to enhance interlayer interactions. An issue commonly encountered in VACNT processing is the tendency for nanotubes to bundle together due to the wet process. This bundling, induced by the liquid zip effect, occurs when small amounts of water cause CNTs to adhere to each other, hindering their vertical alignment. Murata et al. addressed this bundling problem by synthesizing wavy CNTs.^[^ ^181^ ^]^ Their approach involves determining the final density and curvature of the nanotubes by dividing the length of the CNT by the height of the VACNT forest. At relatively low densities, the squeezing effect during CNT growth is reduced, resulting in a curved, wavy shape. Following this, they employed a conventional impregnation‐drying‐H_2_ reduction method to load platinum particles and used a low surface tension ethanol solution to prevent liquid‐induced bundling of the CNTs. Similarly, for ionomer loading, they utilized impregnation, carefully controlling the moisture content of the solution to minimize the liquid zip effect.^[^ ^212^ ^]^ To assemble the MEA, a conventional CCM‐DT process was employed to transfer the prepared catalytic electrodes (**Figure** **17**a). In the LSV test, the mass activity of Pt/VACNT was calculated to be 316 A g^−1^_Pt_, which is 113 A g^−1^_Pt_ higher than the conventional Pt/C electrode (Figure 17b). In addition, a CV test was performed and the ECSA for Pt/VACNT was calculated to be 77 m^2^ g^−1^_Pt_, slightly lower than the 81 m^2^ g^−1^_Pt_ for Pt/C. The analysis suggested that the lower platinum load resulted in a lower ECSA (Figure 17c). In the current–voltage test, which the authors conducted in two separate surface area MEAs, the current density was 2.6 A cm^−2^ at 0.6 V, and the power output per platinum unit mass was 10.4 kW g^−1^, higher than the 8 kW g^−1^ standard given by DOE 2017 (Figure 17d). The morphological analysis implied that the VACNT still has a vertical morphology at the macroscopic level, but a wavy morphology at the microscopic level (Figure 17e). Figure 17f further demonstrates the greater porosity of Pt/VACNT compared to Pt/C catalysts. Remarkably, the wavy VACNT exhibits enhanced mechanical flexibility, implying improved compatibility and contact with rough microporous layer or macroporous support surfaces, particularly under compressive conditions. From the perspective of interlayer mechanical adhesion, the wavy VACNT facilitate improved local contact with the gas diffusion layer and proton exchange membrane. Compared to strictly vertical structures, the slightly curved ends expand the contact surface area, while the vertical morphology and spacing in the central region form the basis for maintaining low interlayer mass transfer resistance.

Regarding the issue of interlayer contact at the gas diffusion layer/catalyst layer interface, it's evident that the perpendicular orientation of VACNT to the proton exchange membrane and gas diffusion layer does not fully maximize contact with the carbon paper, leaving a significant portion of the area unable to form an effective electron pathway. This limitation is inherent in allotropic VACNT, followed by appliqué transfer. Murata et al. proposed a solution involving the controlled compressive stress of the MEA assembly, causing flexible VACNT tips to bend and establish better contact with the microporous layer.^[^ ^181^ ^]^ However, the development of in situ VACNT growth on microporous layer surfaces would offer greater advantages. Earlier efforts by Du et al. involved in situ VACNT growth on carbon paper, while Fontana et al. more recently proposed in situ growth of VACNT on a microporous layer.^[^ ^93^, ^213^ ^]^ However, challenges arise due to the ultra‐high temperature operation of the CVD process and the reducing atmosphere of H_2_, leading to substrate pyrolysis and requiring further processing of metal catalyst nanoparticles. Fontana et al. employed a three‐layer catalyst scheme to protect the carbon substrate. Despite efforts, satisfactory results have not been achieved. While CNT can be grown perpendicular to carbon fibers at the micro level, macro‐level structures fail to form a perpendicular orientation to the proton exchange membrane due to the disordered nature of carbon fibers. Moreover, the lack of single‐sided loading techniques results in later CNT growth on the unfavorable side, wasting numerous CNT structures. In summary, current microporous layer‐based in situ VACNT growth methods remain unsatisfactory, necessitating the development of new techniques to increase the contact surface between VACNT and microporous layer.

While VACNT demonstrates remarkable catalytic capabilities, its effectiveness hinges largely on achieving a highly ordered and vertically aligned morphology. The application of magnetron sputtering for loading platinum particles typically restricts deposition to the outer side of the VA‐MWCNT, resulting in significant wastage of the inner tube wall. Additionally, the hydrophobic characteristics of VACNT contribute to ionomer accumulation during subsequent spray loading, resulting in an overly thick layer of ionomer. Solution‐based wet processes can cause VACNTs to agglomerate together during the drying phase due to capillary effects, leading to a loss of vertically aligned structure. Although this “liquid zip” effect may reduce the forest structure, some efforts have exploited it to create VACNT table patterns.^[^ ^212^, ^214^ ^]^ Regarding synthesis technology, the predominant VACNT preparation method relies on CVD, which operates at temperatures typically ranging from 1000 to 1200 °C. This high‐temperature process results in substantial energy consumption and presents significant challenges for commercialization.^[^ ^120^ ^]^ Although plasma‐assisted CVD techniques have been reported to lower the temperatures to around 600 °C, their application remains limited to laboratory scales.^[^ ^180^ ^]^ Furthermore, previous VACNT synthesis involved ectopic growth on template materials followed by transfer onto the proton exchange membrane using applique transfer, but the drawbacks associated with this ex‐situ growth approach underscore the need for in situ VACNT growth techniques on the gas diffusion layer.^[^ ^93^ ^]^ In summary, the current challenges of VACNT schemes can be categorized as follows: i) issues with morphological control of VACNT, ii) challenges related to wet process agglomeration, iii) difficulties in achieving effective gas diffusion layer/catalyst layer interlayer contact, iv) the need to develop low‐temperature growth and liquid phase dispersion techniques.

Interdisciplinary material design concepts, such as those from biotechnology and sensor technology,^[^ ^215^ ^]^ offer inspiration for the creation of low‐cost CNT dispersion methods. Among these, dielectrophoresis stands out as a technique capable of arranging CNTs into structurally ordered arrays. For instance, Ahadian et al. utilized dielectrophoresis to position CNTs within gelatin methacrylate (GelMA) hydrogels, leading to an increased number of functional myofibers. Notably, the application of electrical stimulation in the direction of the aligned CNTs resulted in a more significant enhancement in myogenic gene and protein expression.^[^ ^216^ ^]^ Similarly, Noh et al. achieved the oriented arrangement of CNTs between two L‐shaped Cu sheets.^[^ ^217^ ^]^ The high conductivity of water‐based suspensions, combined with the anisotropic conductivity of CNTs, facilitates the large‐scale alignment of CNTs. However, achieving this alignment necessitates the use of suspensions with low surface tension and high conductivity due to the liquid shear effect. Despite these advancements, there is currently a lack of studies investigating the application of dielectrophoretically aligned CNTs in fuel cell systems, leaving the feasibility of such an approach uncertain.

## 4 Conclusion

In summary, this review presented the basic principles of hydrogen fuel cells, including the structure of the MEA, fuel cell operating process, ORR mechanism, and fuel cell characterization techniques. The review also examined the factors contributing to poor interlayer contact based on the existing improvable interlayer interactions, which have received less attention in LT‐PEMFC. Additionally, this review highlights the significant potential of CNTs in enhancing the performance and durability of PEMFCs. The integration of CNTs, particularly in the form of VACNT structures, presents a promising pathway for improving electron and mass transport within the MEA. This advancement addresses critical challenges such as catalyst layer design, material durability, and cost‐effectiveness, which have historically hindered the commercialization of PEMFC technology. The review underscores the importance of understanding and optimizing interlayer interactions within the MEA to achieve sustainable and high‐performing fuel cell systems.

CNTs and their structural variants demonstrate significant potential in enhancing interlayer mechanical adhesion, conductive networks, mass transfer, and thermal management within MEAs. However, several critical issues remain unresolved: 1) Achieving efficient surface modification to balance conductivity and mass transfer performance without compromising the CNT framework; 2) The high cost and limited scalability of high‐temperature CVD synthesis and transfer processes for VACNTs; 3) Unclear mechanisms governing the mechanical stability and water management at the catalyst layer‐membrane interface under long‐term dynamic operating conditions; 4) Lack of multiscale models spanning from the nanoscale to the stack scale to guide structural optimization.

To address these challenges, future research may focus on the following directions: Firstly, developing low‐cost, controllable and mild CNT surface modification methods to balance electrochemical activity with structural integrity; secondly, exploring transferable processes and hybrid material strategies based on VACNTs to reduce costs and enhance applicability; thirdly, combining in situ characterization with multiscale simulations to elucidate interfacial water management and mechanical failure mechanisms; Fourth, conducting long‐term durability testing and engineering validation at the MEA and stack levels to establish a closed‐loop optimization between experimental and theoretical approaches.

## Conflict of Interest

The authors declare no conflict of interest.
